# The mesencephalic locomotor region sends a bilateral glutamatergic drive to hindbrain reticulospinal neurons in a tetrapod

**DOI:** 10.1002/cne.23911

**Published:** 2015-11-07

**Authors:** Dimitri Ryczko, Francois Auclair, Jean‐Marie Cabelguen, Réjean Dubuc

**Affiliations:** ^1^ Groupe de Recherche sur le Système Nerveux Central, Département de neurosciencesUniversité de MontréalMontréalQuébecCanada; ^2^ INSERM U862 ‐ Neurocentre MagendieMotor System Diseases TeamBordeaux CedexFrance; ^3^ Groupe de Recherche en Activité Physique Adaptée, Département des sciences de l'activité physiqueUniversité du Québec à MontréalQuébecCanada

**Keywords:** locomotor circuits, brainstem, salamander, conservation

## Abstract

In vertebrates, stimulation of the mesencephalic locomotor region (MLR) on one side evokes symmetrical locomotor movements on both sides. How this occurs was previously examined in detail in a swimmer using body undulations (lamprey), but in tetrapods the downstream projections from the MLR to brainstem neurons are not fully understood. Here we examined the brainstem circuits from the MLR to identified reticulospinal neurons in the salamander *Notophthalmus viridescens*. Using neural tracing, we show that the MLR sends bilateral projections to the middle reticular nucleus (mRN, rostral hindbrain) and the inferior reticular nucleus (iRN, caudal hindbrain). Ca^2+^ imaging coupled to electrophysiology in in vitro isolated brains revealed very similar responses in reticulospinal neurons on both sides to a unilateral MLR stimulation. As the strength of MLR stimulation was increased, the responses increased in size in reticulospinal neurons of the mRN and iRN, but the responses in the iRN were smaller. Bath‐application or local microinjections of glutamatergic antagonists markedly reduced reticulospinal neuron responses, indicating that the MLR sends glutamatergic inputs to reticulospinal neurons. In addition, reticulospinal cells responded to glutamate microinjections and the size of the responses paralleled the amount of glutamate microinjected. Immunofluorescence coupled with anatomical tracing confirmed the presence of glutamatergic projections from the MLR to reticulospinal neurons. Overall, we show that the brainstem circuits activated by the MLR in the salamander are organized similarly to those previously described in lampreys, indicating that the anatomo‐physiological features of the locomotor drive are well conserved in vertebrates. J. Comp. Neurol. 524:1361–1383, 2016. © 2015 The Authors The Journal of Comparative Neurology Published by Wiley Periodicals, Inc.

A remarkably conserved feature relative to the supraspinal control of locomotion in vertebrates is the presence of locomotor centers dedicated to initiating and controlling locomotor output. One such locomotor center is located at the junction between the midbrain and the hindbrain. It was first discovered in cats by a Russian group in the 1960s and named the mesencephalic locomotor region (MLR; Shik et al., 1966). This region was shown to be present in all vertebrates tested from lampreys to mammals (for review, see Ryczko and Dubuc, 2013). Anatomical studies provided evidence that the MLR projects down to reticulospinal (RS) neurons, which in turn activate the spinal central pattern generator (CPG) for locomotion (Steeves and Jordan, 1980, 1984; Shefchyk et al., 1984; Garcia‐Rill and Skinner, 1987a, 1987b; Bachmann et al., 2013). Interestingly, stimulation of the MLR on one side elicits bilateral, symmetrical locomotor movements in all vertebrate species examined as of now. In lampreys, the supraspinal circuitry responsible for the transformation a unilateral stimulation into a bilaterally symmetrical locomotor command was described in detail (Brocard et al., 2010): the MLR sends symmetrical inputs down to RS neurons on both sides, ensuring a symmetrical descending drive to the spinal cord. This was proposed as accounting for the symmetry of locomotor movements. The MLR inputs to RS cells were shown to be both glutamatergic and cholinergic (i.e., nicotinic) (Brocard and Dubuc, 2003, Le Ray et al., 2003). In addition, the MLR activates a group of muscarinoceptive cells in the brainstem, that in turn send an additional excitatory drive to RS cells, allowing swimming activity to reach higher speeds (Smetana et al., 2010).

In tetrapods, the downward connectivity from the MLR is still unknown. The pioneering study of Orlovsky (1970) indicated that the MLR provides inputs to RS cells on both sides, but the symmetry of these inputs on left and right sides was not determined. More recently, studies in mice showed that around 20% of Lhx3‐Chx10 glutamatergic RS neurons are excited by MLR stimulation (Bretzner and Brownstone, 2013). Part of the MLR input may be glutamatergic, as optogenetic stimulation of glutamatergic neurons in the MLR elicits locomotion in vivo in mice (Lee et al., 2014). In line with this, the local application of glutamate over the reticular formation in cats was shown to elicit locomotion (Noga et al., 1988) and to elicit excitatory responses in reticulospinal neurons (Tebecis, 1973; Greene and Carpenter, 1985). The contribution of serotonin and noradrenalin is unlikely to be needed because MLR stimulation is efficient even after depletion of these systems in cats (Steeves et al., 1980). However, the mechanisms by which the MLR controls RS neurons are not fully resolved in tetrapods.

Salamanders are an ideal model to examine the organization of the MLR locomotor drive to RS neurons in tetrapods. Salamander RS neurons are accessible for anatomical, electrophysiological, and Ca^2+^ imaging studies in controlled in vitro conditions with the brainstem circuitry intact. The MLR of salamanders was recently identified (Cabelguen et al., 2003) and it was shown that it controlled both locomotor modes in these animals. Low stimulation intensities evoke stepping, whereas higher stimulation intensities evoke swimming. The salamander MLR colocalizes with a cluster of cholinergic cells (Cabelguen et al., 2003), as in lampreys (Le Ray et al., 2003) and mammals (Garcia‐Rill et al., 1987). As in other vertebrates, it is presumed that the MLR of salamanders projects to hindbrain RS neurons (Naujoks‐Manteuffel and Manteuffel, 1988; Sanchez‐Camacho et al., 2001; Hubbard et al., 2010). Extracellular recordings were made in the hindbrain reticular formation of salamanders following MLR stimulation (Bar‐Gad et al., 1999; Kagan and Shik, 2004) and the reticular formation was shown to be activated during stepping and swimming in animals chronically implanted with microelectrodes (Lowry et al., 1996; Hubbard et al., 2010). Around one‐quarter of the recorded cells that were activated showed an increased activity during swimming compared to stepping (Lowry et al., 1996).

Here we used Ca^2+^ imaging and neural tracing experiments to examine the mechanisms by which MLR stimulation activates RS neurons in the salamander (*Notophthalmus viridescens*). We show that a unilateral activation of the MLR evoked a bilateral activation of RS neurons in the middle (mRN) and inferior (iRN) reticular nuclei. Glutamatergic neurotransmission is involved. We also demonstrate that when increasing the intensity of the MLR stimulation, the RS responses increased both in the mRN and iRN. Overall, we show that the locomotor drive from the MLR to RS cells is strikingly similar to that previously reported in lampreys, suggesting that the structure and organization of brainstem locomotor circuits were conserved in vertebrates.

## MATERIALS AND METHODS

### Ethics statement

All procedures conformed to the guidelines of the Canadian Council on Animal Care and were approved by the animal care and use committees of the Université de Montréal (QC, Canada) and Université du Québec à Montréal (QC, Canada). Care was taken to minimize the number of animals used and their suffering.

### Animals

Experiments were performed on 45 fully metamorphosed salamanders (*Notophthalmus viridescens)* of either sex with snout–vent length (SVL) ranging from 40 to 50 mm. They were purchased from Connecticut Valley Biological Supply (MA, USA) or Boreal Science (ON, Canada). The animals were kept in aerated water at 20°C and fed once or twice a week with frozen bloodworms or *Daphnia*.

### Surgical procedures

The dissection procedure was the same for both physiological and anatomical experiments. The animals were anesthetized by immersion in a 0.2% aqueous solution of tricaine methanesulphonate (MS‐222, 200 mg/L) and then transferred to a cold oxygenated (8–10°C, 100% O_2_) Ringer's solution with the following composition (in mM): 130 NaCl, 2.1 KCl, 2.6 CaCl_2_, 1.8 MgCl_2_, 4 HEPES, 4 dextrose, and 1 NaHCO_3_ (pH 7.4). The animals were decapitated and all soft tissue around the cranium was removed. The brain and the first spinal cord segments were exposed dorsally. A total of 13 preparations were used for anatomical experiments, and 32 for physiological experiments.

### Anatomical tracing

**Figure 1:**
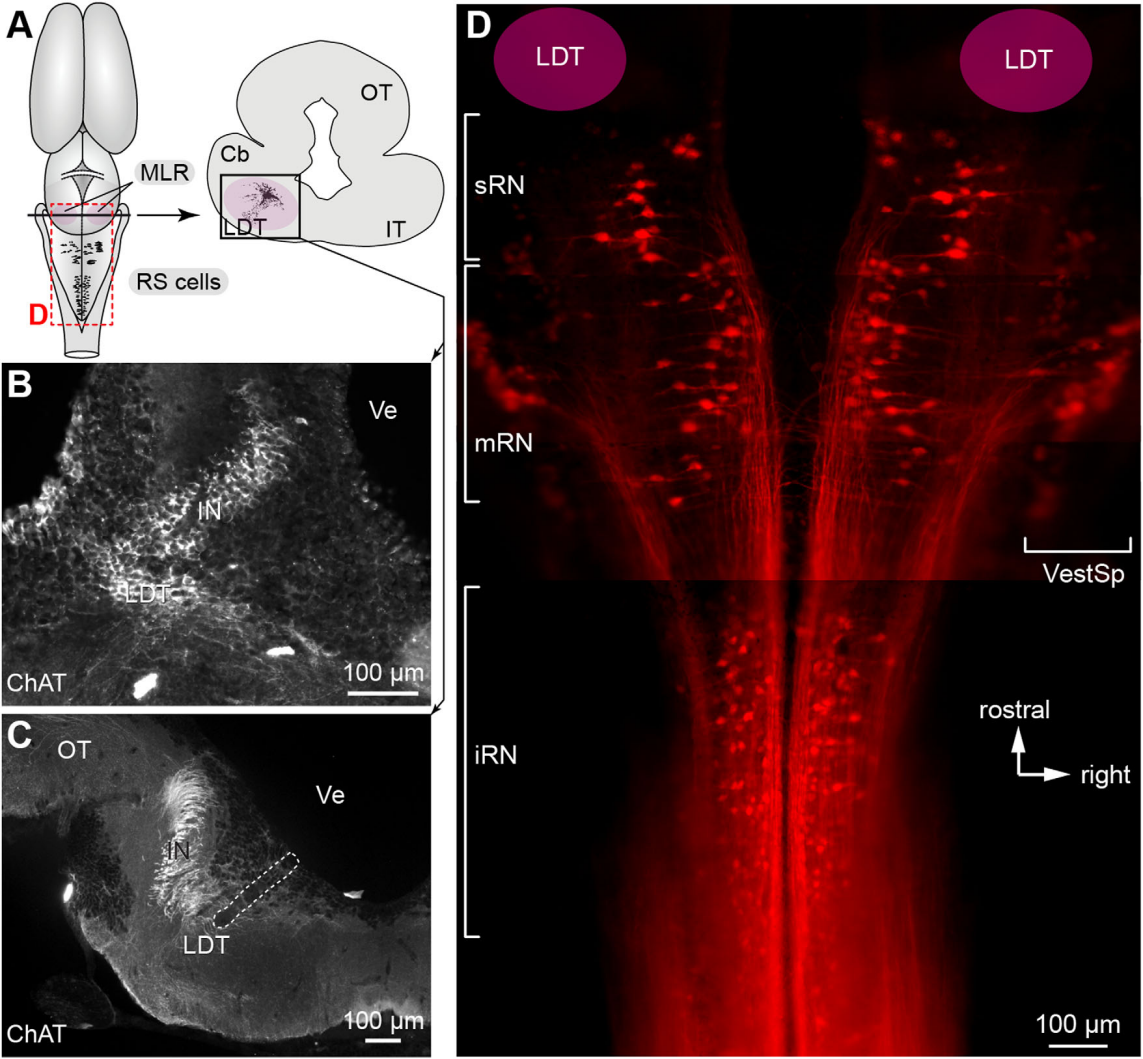
The mesencephalic locomotor region (MLR) and reticulospinal (RS) neurons in *Notophthalmus viridescens*. **A**: Left: Schematic dorsal view of the salamander brain. The MLR as well as the rhombencephalic RS neurons of the middle reticular nucleus (mRN) and inferior reticular nucleus (iRN), which were recorded in the present study, are illustrated according to the labeling shown in D. Right: Cross‐section at the level of the MLR. **B**: Photomicrograph illustrating neurons positive for choline acetyltransferase (ChAT, in white) in the MLR. **C**: Cholinergic cells (white) of the laterodorsal tegmental nucleus (LDT) around the MLR stimulation site that was identified by an electrolytic lesion (enclosed by a white dashed region). The stimulation site was identified consistently with a previous study (Cabelguen et al., 2003). Note that a midsagittal dorsal incision was made in the mesencephalon to give access to the MLR, which is located ventrally. **D**: RS cells were retrogradely labeled by an injection of Texas Red Dextran Amine at the level of the first spinal cord segment (*n* = 3). The brain was dehydrated and cleared in methyl salicylate (see Materials and Methods), and photographed as a wholemount under the microscope. The dorsal part of the mesencephalon was removed to improve visibility. The illustration was obtained by merging five photomicrographs taken at different focal planes and at different rostrocaudal levels in the brainstem. The location of the LDT, corresponding to the MLR stimulation site (Cabelguen et al., 2003), is illustrated. Rostrally, the superior reticular nucleus (sRN) located in the isthmus is indicated. More caudally, the mRN and the iRN are indicated. On both sides, the vestibulospinal neurons (VestSp) are visible. The RS cell nomenclature used is consistent with previous characterizations of brainstem descending neurons in amphibians (Naujoks‐Manteuffel and Manteuffel, 1988; Sanchez‐Camacho et al., 2011). Cb, cerebellum; IN, isthmic nucleus; IT, isthmic tegmentum; OT, optic tectum; Ve, ventricule.

The location of the middle (mRN) and inferior (iRN) reticular nuclei (Naujoks‐Manteuffel and Manteuffel, 1988; Sanchez‐Camacho et al., 2001) was first verified by injections of a tracer (Texas Red Dextran Amine, MW 3,000 Da) at the first spinal segment (*n* = 3). The two nuclei were visualized in wholemount brainstems fixed and made transparent by dehydration in graded alcohols followed by immersion in methyl salicylate (Fig. 1D).

In another set of experiments that relied on the above results for the precision of injections, the anatomical tracer, biocytin, was injected in either the mRN (*n* = 4) or the iRN (*n* = 5) to retrogradely label cells of the MLR that project to those nuclei. A similar procedure was used successfully on several occasions by us in lampreys (e.g., Antri et al., 2008; Derjean et al., 2010; Gariépy et al., 2012a, 2012b; Ryczko et al., 2013). A complete transverse section of the tegmentum was carried out unilaterally with a microscalpel at the level of the rostral portion of each nucleus. The gap created was filled with crystals of biocytin for 10 minutes, allowing the tracer to fill the cut axons. The brain was then rinsed thoroughly with Ringer's and transferred to a chamber continuously perfused with oxygenated Ringer's at room temperature for 3–4 hours to allow the retrograde transport of the tracer. The whole preparation was then fixed for 24 hours by immersion in a solution of 4% paraformaldehyde / 0.5% glutaraldehyde in phosphate‐buffered saline (PBS) for 24 hours. It was then cryoprotected in a phosphate‐buffered solution containing 20% sucrose. The next day the brain was sectioned on a cryostat at 40 μm or 25 μm thickness, and the sections collected in PBS (0.1M, pH 7.4, NaCl 0.9%).

### Glutamate immunofluorescence

Freshly cut 40‐μm sections were rinsed with PBS 3 times for 10 minutes each. The sections were then incubated in a solution of 5% normal goat serum in PBS for 60 minutes. The same solution was used to dilute the primary and secondary antibodies. The sections were incubated with a rabbit antiglutamate antibody (diluted 1:3,000, IG1007, lot 3603, ImmunoSolution, Australia, RRID: AB_10013224) overnight at room temperature. The following day the sections were rinsed 3 times 10 minutes each in PBS and incubated with a goat antirabbit‐Alexa Fluor 594 antibody (diluted 1:500, A11012, Invitrogen, La Jolla, CA) for 120 minutes. The sections were then rinsed 3 times for 10 minutes each with PBS and mounted on microscope slides with Vectashield containing DAPI (H‐1200, Vector, Burlingame, CA). Omitting the primary antibody from the procedure resulted in total absence of labeling. The IG1007 antibody has been used successfully to label glutamatergic neurons in lamprey brains (Barreiro‐Iglesias et al., 2008; Villar‐Cerviño et al., 2011). According to the supplier data sheet, the polyclonal rabbit antiglutamate antibody was raised against glutamate that has been conjugated to a carrier protein using glutaraldehyde. Its specificity toward glutamate was confirmed by the supplier following tests in monkey, cat, rat, and mouse tissues where the antibody successfully localized glutamate in glutamatergic retinal photoreceptors and cortical pyramidal cells. The dotblots did not yield significant immunoreactions against a variety of amino acid conjugates such as aspartate. Furthermore, western blots revealed no staining of lamprey brain proteins extracts (Barreiro‐Iglesias et al., 2008; Villar‐Cervino et al., 2011). In addition, lamprey brain regions containing neurons labeled with this antiglutamate antibody also contained neurons expressing high levels of the vesicular transporter for glutamate (VGLUT) mRNA (Villar‐Cervino et al., 2011). Finally, the staining obtained with this antibody was reported to be similar to that obtained with a mouse monoclonal antiglutamate antibody in sections of lamprey brain and retina (Fernandez‐Lopez et al., 2012).

### Electrophysiological experiments

The MLR was stimulated using homemade glass‐coated tungsten microelectrodes (0.7–3.1 MΩ, 10‐35 μm exposed tip). Stimulation was delivered using a Grass Instrument (Quincy, MA) S88 stimulator coupled to a Grass PSIU6 photoelectric isolation unit. The location of stimulation site was identified on the basis of previous anatomical and physiological experiments performed in the same species (Cabelguen et al., 2003). The location of the stimulation site was verified histologically after performing an electrolytic lesion at the end of the experiment (5 seconds, DC current, 10 μA) combined with immunofluorescence against choline acetyltransferase (ChAT, see below). Square pulses of 2 ms duration were applied either in single pulse mode or repetitively using 10‐second trains with a frequency of 5 Hz. The stimulation intensities ranged from 2 to 15 μA for single pulse, and from 1 to 8 μA for train stimulation. Extracellular recordings of the descending RS axons were also performed as described elsewhere (e.g., Ryczko et al., 2013) to monitor the activation of the final common descending pathway for locomotion. The tip of a glass micropipette (tip diameter around 5 μm) filled with Ringer's solution was placed on the rostral stump of the cut spinal cord at the level of the first segment. Signals were amplified (bandwidth 100–500 Hz, model 1800, A‐M Systems, Everett, WA) and digitized with a Digidata 1200 series interface coupled with Clampex 9.0 software (Axon Instruments, Burlingame, CA). Stimulation timing was recorded in a separate channel. To provide better visibility of the electrophysiological responses, the stimulus artifacts were removed using Spike2 5.19 software (Cambridge Electronic Design, Cambridge, UK).

### Ca^2+^ imaging

The imaging procedure was similar to that previously used by us (Viana Di Prisco et al., 1997; Brocard et al., 2010; Derjean et al., 2010). The brain rostral to the mesencephalon was removed following a transverse section. The RS neurons were retrogradely labeled by placing crystals of Ca^2+^ Green dextran amine (MW, 3,000 Da, Invitrogen, La Jolla, CA) at the level of the first segment of the spinal cord after a transection. The brain was then placed into a cold and oxygenated chamber for 18–24 hours to allow the dye to retrogradely label RS neurons before imaging. The following day, a dorsal midsagittal transection was performed in the mesencephalon to provide access to the MLR. The preparation was pinned down dorsal side up to the bottom of a recording chamber covered with Sylgard (Dow Corning, Midland, MI) and perfused with oxygenated Ringer's solution (4 mL/min) cooled to a temperature of 8–10°C. A stimulation electrode was placed unilaterally in the MLR. The RS nuclei were easily identified under the microscope on the basis of our RS distribution map (Fig. 1A,D) as well as previous reports describing the distribution of RS cells in salamanders (Naujoks‐Manteuffel and Manteuffel, 1988; Sanchez‐Camacho et al., 2001). An optimal focal plane was chosen for imaging cells in the mRN and/or iRN. To measure the change in fluorescence in RS neurons, regions of interest were manually delineated around the RS cell bodies labeled with the Ca^2+^ dye. The Ca^2+^ responses of RS neurons to MLR stimulation were acquired at the rate of two images per second with a Nikon epifluorescent microscope coupled with a CCD video camera (Photometrics CoolSNAP HQ, Roper Scientific, Tucson, AZ). The Ca^2+^ responses were expressed as the relative change in fluorescence (ΔF/F). For each cell, baseline was defined as the averaged fluorescence of the cell body before stimulation. In two experiments, the location of the stimulation site was verified histologically (see ChAT immunofluorescence, below). Data analysis was carried out using Metafluor (Universal Imaging, West Chester, PA), Clampfit (Molecular Devices, Palo Alto, CA) and MatLab (MathWorks, Natick, MA). All quantifications of RS responses were done by calculating the area under the curve from the beginning of the response to the return to baseline using the area calculation script in Clampfit (Molecular Devices). The areas are expressed in ΔF/F × s.

### Drug application

All drugs were obtained from Sigma (St. Louis, MO) and diluted to their final concentration in Ringer's. In some experiments, the AMPA/Kainate antagonist 6‐cyano‐7‐nitroquinoxaline‐2,3‐dione (CNQX, 25 μM), the NMDA antagonist (2R)‐amino‐5‐phosphonovaleric acid (AP5, 100 μM) were bath‐applied. In other experiments, a Ringer's solution containing CNQX (1 mM) and AP5 (500 μM) were microinjected locally onto RS neurons, and a Ringer's solution containing D,L‐glutamate (2.5 mM) was injected onto RS neurons or in the MLR. The microinjection procedure was the same as previously described (Brocard and Dubuc, 2003; Le Ray et al., 2003; Brocard et al., 2005, 2010; Derjean et al., 2010; Smetana et al., 2010; Gariépy et al., 2012a, 2012b; Ryczko et al., 2013). Briefly, drugs were microinjected through a glass micropipette (diameter 10–20 μm) by applying pressure pulses (3–4 PSI) of various durations (20–100 ms) with a Toohey microinjection system (Fairfield, NJ). The injected volumes were estimated by measuring the volume of a droplet ejected in the air following a single pressure pulse multiplied by the number of pressure pulses used per microinjection (see Le Ray et al., 2003; Brocard et al., 2005; Ryczko et al., 2013). The number of moles ejected was calculated for each drug. For all drug applications, the drugs were washed out for a period of 60 to 180 minutes.

### ChAT immunofluorescence

To confirm the location of stimulation electrodes in electrophysiological and Ca^2+^ imaging experiments (*n* = 2), immunofluorescence against ChAT was carried out a posteriori to visualize the cholinergic cells of the laterodorsal tegmental nucleus (LDT) (Fig. 1A,C). An additional intact preparation was used to illustrate the cluster of cholinergic neurons in the LDT (Fig. 1B). At the end of the physiological experiments, the preparations were fixed by immersion in a solution of 4% paraformaldehyde in PBS for 24 hours, followed by immersion in a phosphate‐buffered solution containing 20% sucrose. The next day the brain was removed and sectioned transversely at 25 μm thickness on a cryostat, the sections collected on ColorFrost Plus microscope slides (Fisher Scientific, Pittsburgh, PA) and air‐dried overnight at 37°C on a warming plate. The sections were then rinsed 3 times for 10 minutes each with PBS and incubated for 60 minutes in a solution containing 0.3% Triton X‐100 and 5% normal horse serum in PBS. The same solution was used to dilute the primary and secondary antibodies. The sections were then incubated overnight at 4°C in a goat anti‐ChAT antibody (diluted 1:100, AB144P, lots 2010060 and 1780580, Millipore, Bedford, MA). The following day the sections were rinsed 3 times for 10 minutes in PBS and incubated for 60 minutes in a donkey antigoat‐Alexa Fluor 488 antibody (diluted 1:200, A11055, Invitrogen). The sections were rinsed 3 times for 10 minutes each with PBS and the slides mounted with Vectashield containing DAPI. No labeling was obtained when the primary antibody was removed from the procedure. The AB144P ChAT antibody has been used for many years by different research teams and its selectivity has been well demonstrated in salamanders (Marin et al., 1997; Cabelguen et al., 2003) and in lampreys (Pombal et al., 2001; Le Ray et al., 2003; Quinlan and Buchanan, 2008; Ryczko et al., 2013).

### Epifluorescence microscopy and cell counts

The sections were observed and photographed using an E600 epifluorescence microscope equipped with a DXM1200 digital camera (Nikon, Canada). Combining digital photomicrographs taken with different filter sets and adjusting the levels was done using Photoshop CS5 (Adobe Systems, San Jose, CA). Retrogradely labeled MLR neurons were counted on some preparations (*n* = 3) from the glutamate immunofluorescence experiments (40‐μm thick sections) and on additional animals (*n* = 3) that were injected for this purpose only (25‐μm thick sections). To avoid double counting due to sectioning, and keeping in mind that only relative numbers were sufficient for the purpose here, two approaches were used depending on the section thickness. For 25‐μm sections, all cells on all focal planes were counted on every two sections. For 40‐μm sections, cells were counted from the top surface of each section (using the first available focal plane with a 40× objective), while making sure that all sections were in the same orientation. In both cases, the cell counts obtained represent a significant underestimation of the actual number of labeled cells in each preparation.

### Statistics

Data are expressed as mean ± standard error of the mean (SEM). Statistical analyses were performed using Sigma Plot (Systat, Point Richmond, CA) or MatLab (MathWorks). Statistical differences were assumed to be significant when *P* < 0.05. A two‐tailed paired Student's *t*‐test was used to compare means between two dependent groups. To compare more than two dependent groups, a parametric one‐way analysis of variance (ANOVA) for repeated measures or a nonparametric Friedman ANOVA on ranks for repeated measures were used. Both analyses were followed by a Student‐Newman‐Keuls post‐hoc test. Correlations between variables and their significance as well as 95% confidence intervals were calculated using Sigma Plot (Systat) or the Curve Fitting toolbox (cftool) in MatLab (MathWorks). Figures were made with Adobe Illustrator.

## RESULTS

### Effects of the MLR on RS cells

Stimulation of the MLR on one side is known to generate bilaterally symmetrical locomotor movements and to control the intensity of locomotor output in tetrapods (cat: Shik et al., 1966; guinea‐pig: Marlinsky et al., 1991; rabbit: Musienko et al., 2008; salamander: Cabelguen et al., 2003). The neural mechanisms by which the symmetry occurs are still unresolved and we examined this in salamanders. The MLR was electrically stimulated on one side and the evoked responses were quantified in RS cells of the mRN and iRN (Fig. 1A,D) on both sides. In two preparations, histological analysis was used to confirm that the stimulation site was located among cholinergic cells of the laterodorsal tegmental nucleus (LDT, Fig. 1A–C), a nucleus proposed to be part of the MLR (see Cabelguen et al., 2003). In other vertebrates, the localization of such cholinergic cells corresponds to regions from which locomotion can be elicited (e.g., Le Ray et al., 2003, in lampreys; Garcia‐Rill et al., 1987, in rats; see Ryczko and Dubuc, 2013, for review).

**Figure 2:**
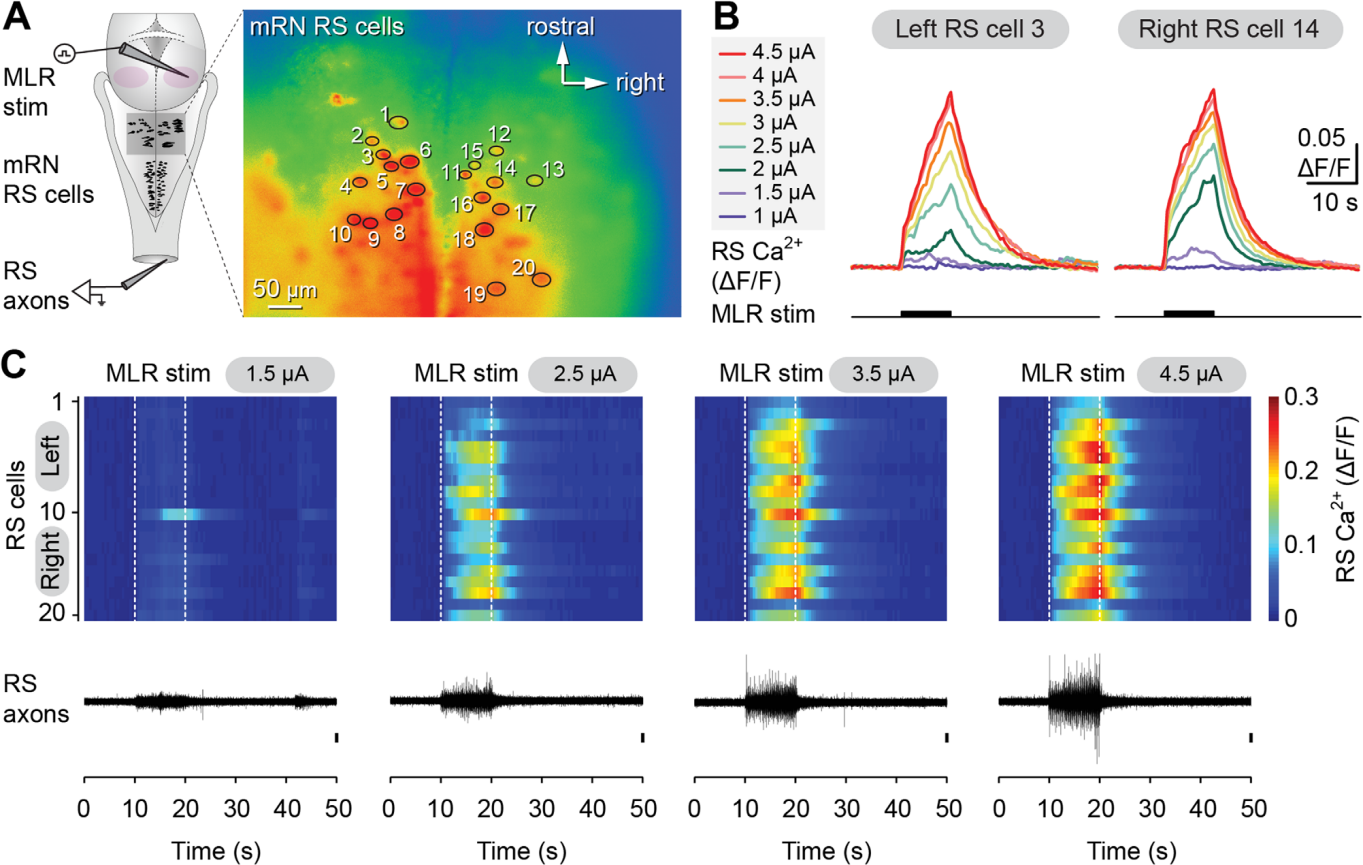
Ca^2+^ responses elicited in reticulospinal (RS) cells of the middle reticular nucleus (mRN) on both sides by MLR stimulation on one side. **A**: Left: Schematic dorsal view of the salamander brain. Right: Ca^2+^ fluorescence at rest of RS cells of the mRN. An electrode was placed over the spinal cord to record the activity of descending RS axons. **B**: Ca^2+^ responses (ΔF/F) in individual RS cells on left and right sides of the brainstem in response to stimulation of the MLR on the right side (1–4.5 μA, 10 seconds train, 5 Hz, 2 ms pulses). **C**: Top: Color plots illustrating the Ca^2+^ responses of the population of RS cells in (A) for increasing MLR stimulation intensities. Each line illustrates the response of individual cells that are numbered from top to bottom: cells 1 to 10 located on the left side of the brain, and cells 11 to 20 on the right side. Onset and offset of MLR stimulation are indicated for each trial with white dotted lines. Warmer colors (red) indicate larger Ca^2+^ responses. Bottom: Electrophysiological activity of descending RS axons recorded simultaneously in the spinal cord. Data from A–C are from the same preparation.

**Figure 3:**
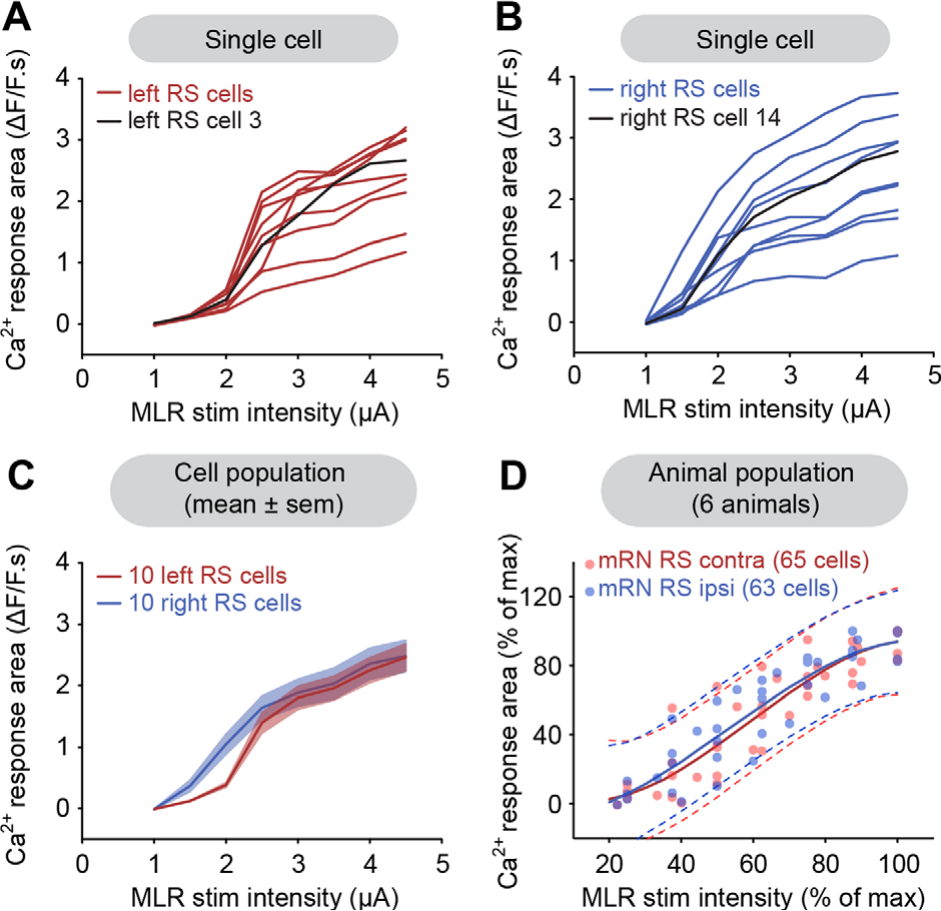
Relationship between the size of the Ca^2+^ responses of individual reticulospinal (RS) cells of the middle reticular nucleus (mRN) and the MLR stimulation intensity. **A,B**: Plots of the size of Ca^2+^ responses vs. stimulation intensity for 10 RS cells in the left (A) and 10 RS cells in the right (B) mRN. The MLR on the right side was stimulated. Each trace represents the responses of an individual cell. The black traces correspond to the cells illustrated in Fig. 2B. **C**: Plot illustrating the size of the Ca^2+^ response (mean ± SEM; pooled data for 10 cells on each side) of RS cells located on the left (red) and right (blue) sides as a function of MLR stimulation intensity. **D**: Relationships between the size of the Ca^2+^ response of ipsilateral (blue dots, *n* = 63 cells) and contralateral (red dots, *n* = 65 cells) RS cells pooled from six preparations and the intensity of MLR stimulation (1–5 μA, 10 seconds train, 5 Hz, 2 ms pulses). Ipsilateral (blue solid line) and contralateral data (red solid line) follow a cubic polynomial function. The dotted lines illustrate the 95% prediction intervals for each function. Data from A–C are from the same preparation as in Fig. 2.

**Figure 4:**
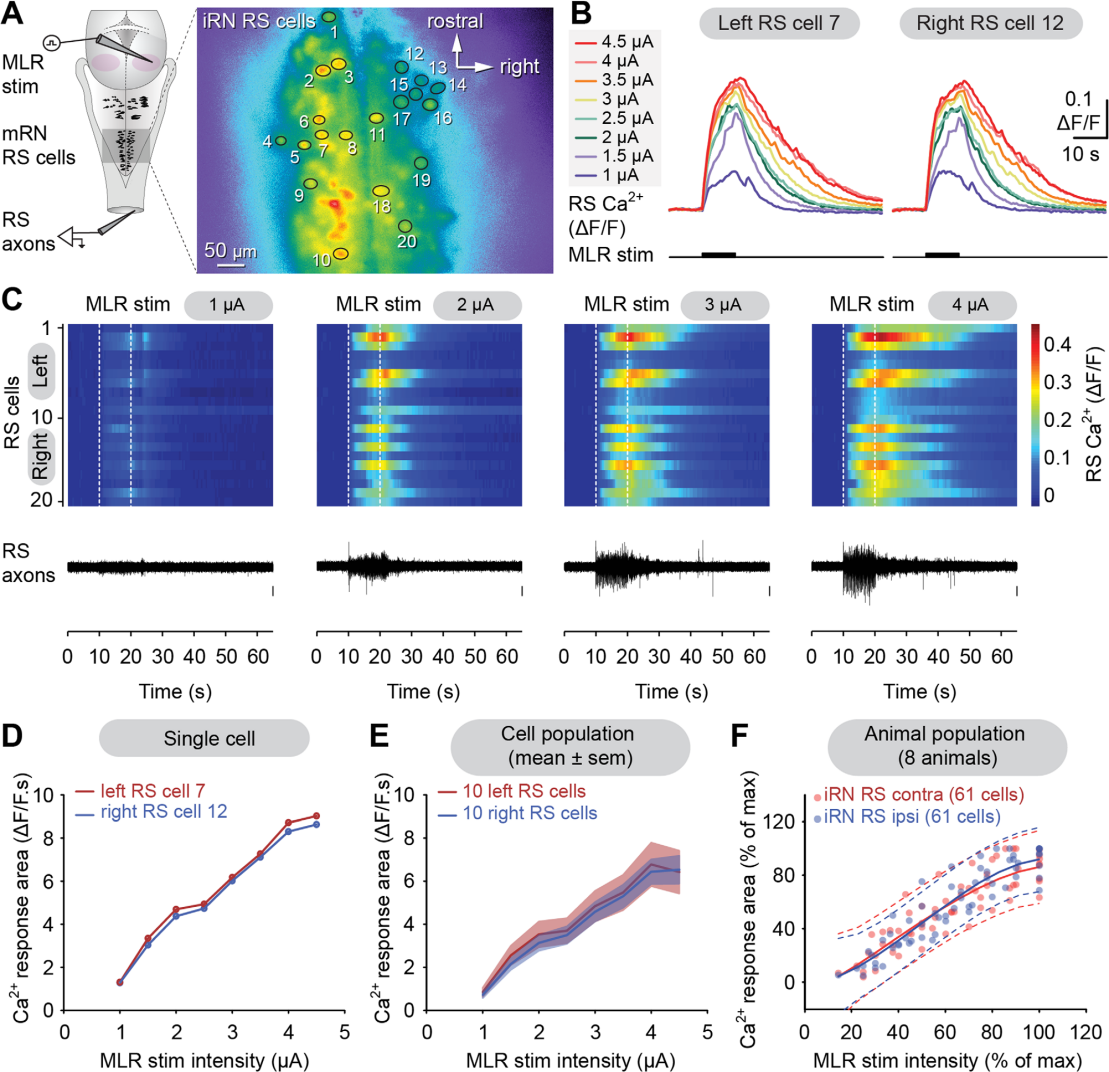
Ca^2+^ responses evoked in reticulospinal (RS) cells of the inferior reticular nucleus (iRN) on both sides by MLR stimulation on one side. **A**: Left: Schematic dorsal view of the salamander brain. Right: Ca^2+^ fluorescence at rest of RS cells of the iRN. An electrode was placed over the spinal cord to record the activity of descending RS axons. **B**: Ca^2+^ responses (ΔF/F) in individual RS cells on left and right sides of the brainstem in response to stimulation of the MLR on the right side (1–4.5 μA, 10 seconds train, 5 Hz, 2 ms pulses). **C**: Top: Color plots illustrating the Ca^2+^ responses of the population of RS cells in (A) for increasing MLR stimulation intensities. Each line illustrates the response of individual cells that are numbered from top to bottom: cells 1 to 10 located on the left side of the brain, and cells 11 to 20 on the right side. Onset and offset of MLR stimulation are indicated for each trial with white dotted lines. Warmer colors (red) indicate larger Ca^2+^ responses. Bottom: Electrophysiological activity of descending RS axons recorded simultaneously in the spinal cord. **D**: Plots of the size of Ca^2+^ response vs. stimulation intensity in two RS cells (left: red trace; right: blue trace) in the iRN. The MLR on the right side was stimulated. The corresponding raw data for these cells are illustrated in B. **E**: Plot illustrating the size of the Ca^2+^ response (mean ± SEM) of RS cells located on the left (red) and right (blue) sides as a function of MLR stimulation intensity. **F**: Relationships between the size of Ca^2+^ response of ipsilateral (blue dots, *n* = 61 cells) and contralateral (red dots, *n* = 61 cells) RS cells pooled from eight preparations and the intensity of MLR stimulation (1–7 μA, 10 seconds train, 5 Hz, 2 ms pulses). Ipsilateral (blue solid line) and contralateral data (red solid line) followed a cubic polynomial function. The dotted lines illustrate the 95% prediction intervals for each function. Data from A–E are from the same preparation.

Increasing the stimulation intensity of the MLR (1–5 μA; 2 ms pulses, 5 Hz, 10 seconds train) elicited Ca^2+^ responses in RS cells of the mRN that grew in size (area of the responses, see Materials and Methods) in a very similar fashion on both sides (raw traces in Fig. 2A–C). Larger Ca^2+^ responses were also associated with larger bursts of discharge recorded in the spinal cord (Fig. 2C), likely resulting from increased spiking activity in the descending RS axons. The Ca^2+^ responses initially grew in size rapidly when increasing stimulation intensity (Fig. 3A–C), to eventually reach a plateau at high stimulation strengths. The results from different preparations were pooled by expressing the responses as a percentage of the maximal Ca^2+^ response. The MLR stimulation intensity was expressed as a percentage of maximal stimulation intensity (*n* = 128 cells recorded from six preparations) as previously done in lampreys (Brocard et al., 2010). The maximal stimulation intensity was defined when the responses were the largest and did not grow any further as the stimulation strength was increased. The relationship between the Ca^2+^ responses and the stimulation intensity followed a cubic polynomial function for ipsilateral (R^2^ = 0.84, *P* < 0.001) as well as for contralateral (R^2^ = 0.84, *P* < 0.001) RS neurons. The two functions were very similar (Fig. 3D, solid lines). To quantify the similarity between the two functions, the 95% prediction intervals were calculated for each function. It was found that 95% (41 of 43 values) of the Ca^2+^ responses in ipsilateral neurons were included in the prediction interval of contralateral neurons, whereas 98% (42 of 43 values) of the Ca^2+^ responses in contralateral neurons were included in the prediction interval of ipsilateral neurons. Similar results were obtained for the iRN. Stimulation of the MLR (1–7 μA; 2 ms pulses, 5 Hz, 10 seconds train) induced very similar Ca^2+^ responses in RS cells on the ipsilateral (R^2^ = 0.86, *P* < 0.001) and contralateral (R^2^ = 0.78, *P* < 0.001) sides (*n* = 122 cells from eight preparations; Fig. 4A–F). The 95% prediction intervals revealed that 97% (59 of 61 values) of the ipsilateral responses were included in the prediction interval of contralateral neurons, whereas 90% (55 of 61 values) of the contralateral responses were included in the prediction interval of ipsilateral neurons. Altogether, these results indicate that a unilateral stimulation of the MLR symmetrically activates RS cells of the mRN and iRN on both sides.

**Figure 5:**
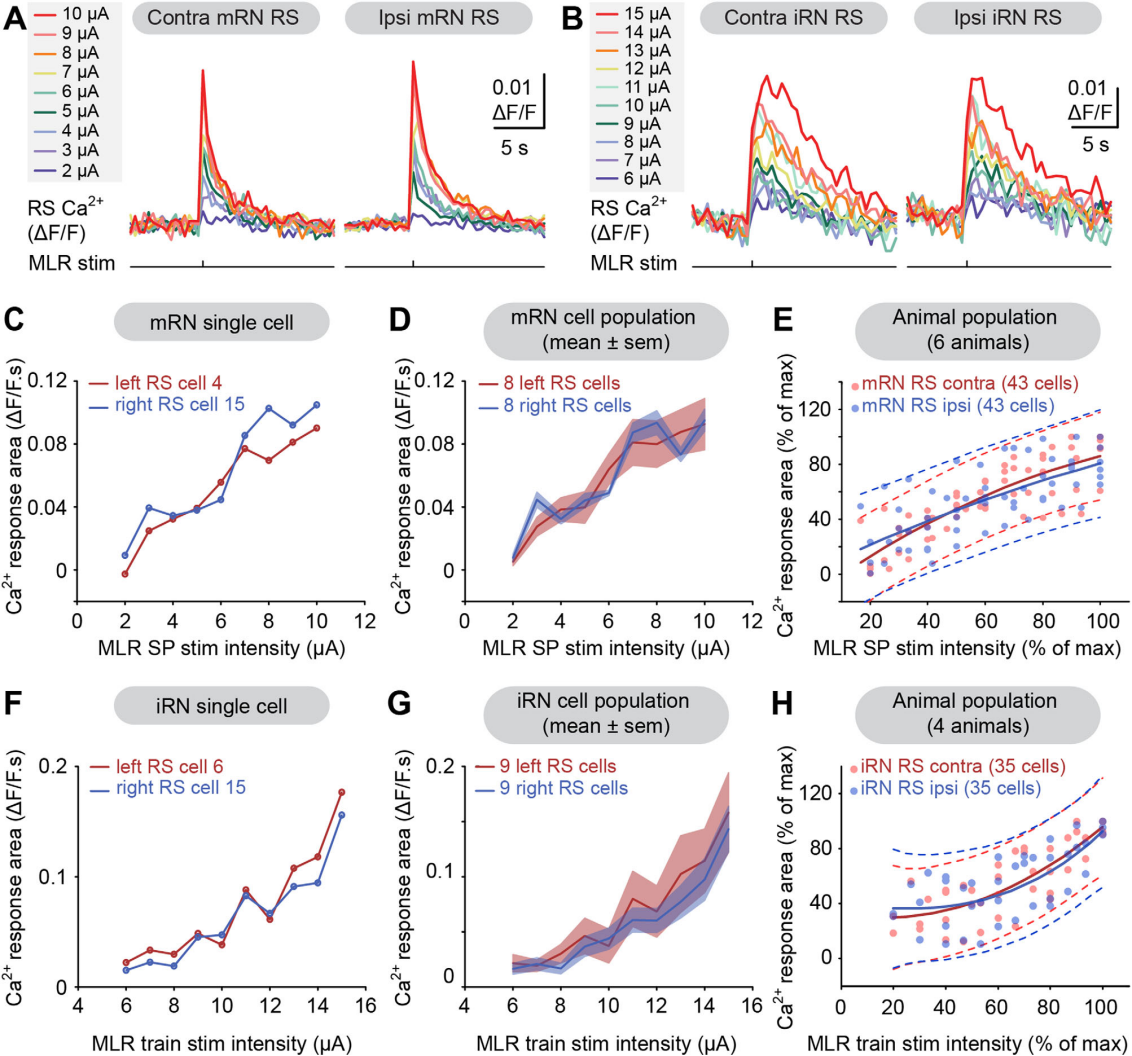
Ca^2+^ responses evoked in reticulospinal (RS) cells of the middle (mRN) and inferior (iRN) reticular nuclei by single pulse (SP) MLR stimulation on one side. **A**: Ca^2+^ responses (ΔF/F) of two RS cells located on the left and right sides of the mRN, in response to SP stimulation of the MLR on the right side (2–10 μA, 2 ms pulses). Note the fast onset of the responses. **B**: Ca^2+^ responses (ΔF/F) of two iRN RS cells located on the left and right sides of the brain in response to SP stimulation of the MLR on the right side (6–15 μA, 2 ms pulses). **C**: Plot illustrating the size of Ca^2+^ responses in two RS cells (left: red trace; right: blue trace) in the mRN vs. the MLR stimulation. The corresponding raw data for these cells are illustrated in A. **D**: Same as in C, but after compiling the responses (mean ± SEM) of eight RS cells on the left (red) and the right (blue). **E**: Relationships between the Ca^2+^ responses of ipsilateral (blue dots, *n* = 43 cells) and contralateral (red dots, *n* = 43 cells) RS cells in the mRN pooled from six preparations and the intensity of MLR stimulation (2–15 μA, 2 ms pulses). Ipsilateral (blue solid line) and contralateral data (red solid line) followed a quadratic polynomial function. The dotted lines illustrate the 95% prediction intervals for each function. **F**: Plot illustrating the size of Ca^2+^ responses in two RS cells (left: red trace; right: blue trace) in the iRN vs. the MLR stimulation. The corresponding raw data for these cells are illustrated in B. **G**: Same as in (F), but after compiling the responses (mean ± SEM) of nine RS cells on the left (red) and the right (blue). **H**: Relationships between the size of the Ca^2+^ responses of ipsilateral (blue dots, *n* = 35 cells) and contralateral (red dots, *n* = 35 cells) RS cells of the iRN pooled from four preparations and the intensity of MLR stimulation (2–15 μA, 2 ms pulses). Ipsilateral (blue solid line) and contralateral data (red solid line) followed a cubic polynomial function. The dotted lines illustrate the 95% prediction intervals for each function. Data from A,C,D and B,F,G are from two different preparations.

Stimulating the MLR with single pulses (2–15 μA, 2 ms pulses) also elicited very clear Ca^2+^ responses in RS cells, suggesting a very powerful excitatory MLR drive to RS cells. Evoked responses to single pulse stimulation were very similar in RS cells of the mRN on the left and right sides (Fig. 5A,C,D). The pooled data from six experiments (86 mRN cells, Fig. 5E) showed that the relationship between the Ca^2+^ responses and the stimulation intensity followed a cubic polynomial function for RS cells located ipsilaterally (R^2^ = 0.52, *P* < 0.001) and contralaterally (R^2^ = 0.71, *P* < 0.001) (blue and red solid lines, respectively). These two polynomial functions were very similar and the 95% prediction intervals revealed that 100% (61 of 61 values) of ipsilateral responses were included in the prediction interval of the contralateral cells, whereas 90% (55 of 61 values) of the contralateral responses were included in the prediction interval of the ipsilateral cells. Similar results were obtained for RS cells in the iRN. Increases in single pulse stimulation intensity of the MLR (2–15 μA, 2 ms pulses) induced similar increases in the responses of RS cells on both sides (Fig. 5B,F,G). The relationships between the stimulation intensity and the size of the responses were also very similar in contralateral (R^2^ = 0.66, *P* < 0.001) and ipsilateral (R^2^ = 0.53, *P* < 0.001) cells (*n* = 70 cells from four preparations), with 100% (40 of 40 values) of ipsilateral responses included in the contralateral prediction interval and vice versa (Fig. 5H). These large responses evoked by single pulse stimulation of the MLR on one side indicate that the MLR provides a powerful, possibly direct excitatory drive to RS cells on both sides.

Electrical stimulation of the MLR not only activates cell bodies, but also axons in the vicinity of the electrode. In two control experiments, electrical stimulation was replaced by chemical stimulation to selectively activate cell bodies in the MLR. D,L‐glutamate was microinjected in the MLR on one side (2.5 mM, 5 seconds train, 2 Hz, 10 pulses, 3–4 PSI, 20–80 ms pulses, 0.36–6.95 nL per microinjection). The injection duration was increased in order to increase the quantity of glutamate (0.91 to 17.38 pmol). The results were very similar to those obtained with electrical stimulation: the area of the Ca^2+^ responses in RS cells of the mRN and iRN progressively increased with the duration of the injection (not illustrated) and followed a similar cubic polynomial relationship.

### Anatomical projections from the MLR to the mRN and iRN

**Figure 6:**
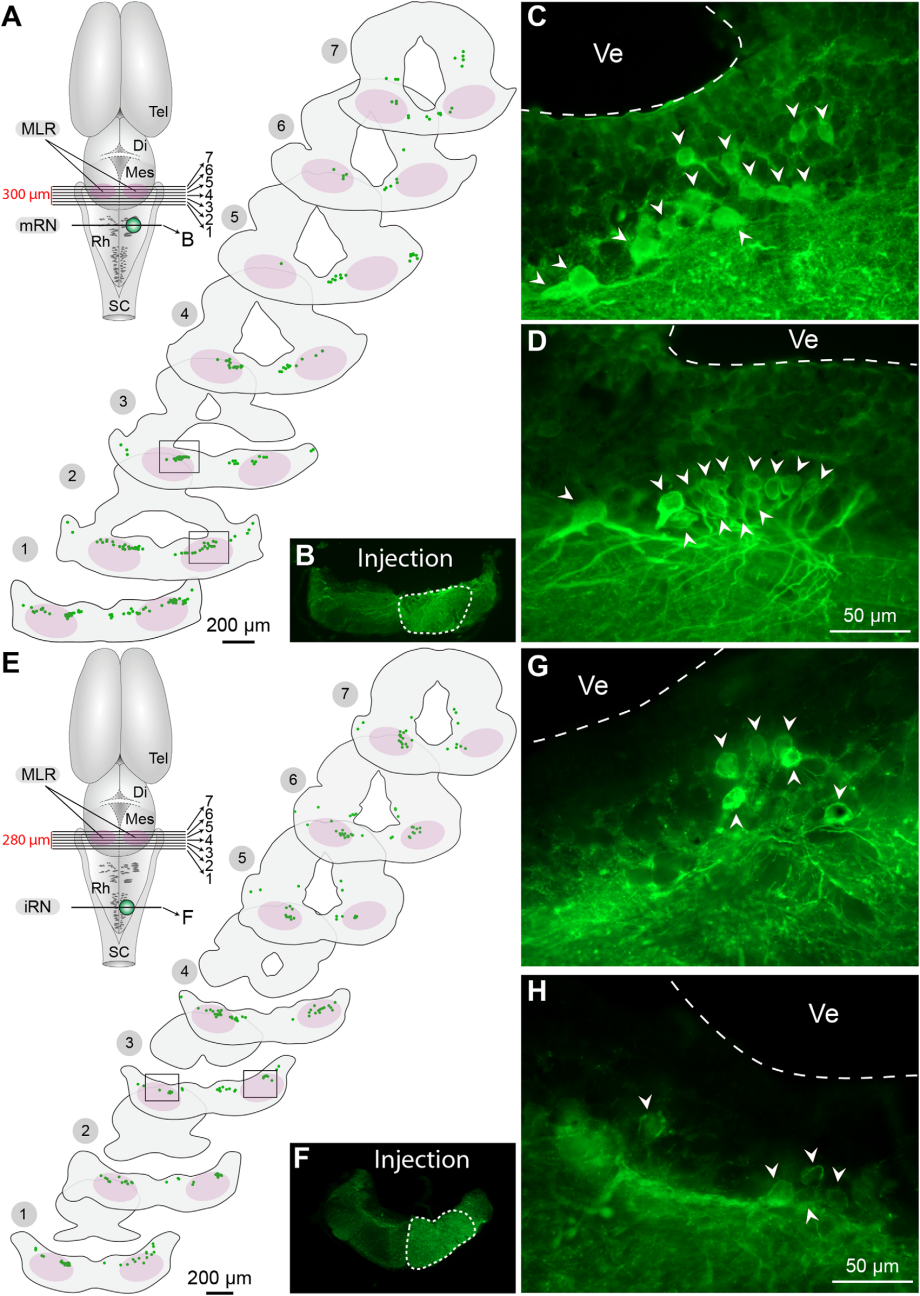
Retrogradely labeled neurons in the isthmic region after unilateral injection of biocytin in the middle (mRN) or inferior reticular nucleus (iRN). **A**: Schematic dorsal view of the salamander brain illustrating the approximate location of cross‐sections 1 to 7, and of the tracer injection (biocytin, green) in the mRN illustrated in (B). On the schematized brain sections, each colored dot represents one neuron labeled with biocytin. The MLR is delineated in pink. In this animal, a total of 206 cells were labeled in the tegmentum of the isthmic region, among which 113 were labeled ipsilateral to the injection site (55% of total), and 93 cells contralaterally (45% of total). **B**: Photomicrograph illustration a biocytin injection site in the mRN (enclosed by a white dashed line). **C,D**: High‐power photomicrographs illustrating examples of retrogradely labeled neurons (arrowheads) located ipsilaterally (C) and contralaterally (D) to the injection site in the mRN. The approximate location of the picture frames is indicated in cross‐sections 2 and 3 in (A). **E**: Same representation as in (A), but this time the tracer biocytin was injected in the iRN on one side. In this animal, a total of 204 cells were retrogradely labeled in the tegmentum of the isthmic region, among which 92 (45% of total) were labeled ipsilaterally and 112 contralaterally (55% of total) to the injection site. **F**: Biocytin injection site on one side in the iRN (enclosed by a white dashed line). **G,H**: High‐power photomicrographs showing retrogradely labeled cells (arrowheads) on both sides of the isthmic region following unilateral injection in the iRN. The approximate location of the picture frames is indicated in cross‐section 3 in (E). Di, diencephalon; Mes, mesencephalon; Rh, rhombencephalon; SC, spinal cord; Tel, telencephalon; Ve, ventricule. A–D, E–H, data are from two different preparations. In these preparations, biocytin was revealed either in blue (Alexa Fluor 350) or in green (Alexa Fluor 488).

Anatomical experiments were then carried out to examine the distribution of cells from the isthmic region projecting down to the reticular formation. Tracer injections in the mRN (Fig. 6A–D) or the iRN (Fig. 6E–H) on one side retrogradely labeled cells on both sides in a region (in pink in Fig. 6A,E) physiologically defined as the salamander MLR (Cabelguen et al., 2003; see also Fig. 1C). The retrogradely labeled cells were located mostly in the isthmic region and rostral hindbrain, in and around the LDT. This region corresponds to the territories referred to as rhombomere 0 (r0, isthmus) and rhombomere 1 (r1) in *Pleurodeles waltl* (Morona and Gonzalez, 2009). A quantitative estimation of the relative importance of the projections from the MLR to ipsilateral or contralateral reticular nuclei was obtained by normalizing cell counts. The number of cells labeled on each side was expressed as a percentage of the total cell count in three preparations injected in the mRN. No significant difference (*P* > 0.05, two‐tailed paired *t*‐test) was found in the number of cells labeled ipsilaterally (53 ± 1% of total cell count, 113 to 182 cells) and contralaterally (47 ± 1% of total cell count, 93 to 169 cells). Similarly, in three other preparations injected in the other reticular nucleus, the iRN, there was no difference in the percentage of cells labeled (*P* > 0.05, two‐tailed paired *t*‐test) in the ipsilateral (53 ± 5% of total cell count, 21 to 95 cells) and the contralateral (47 ± 5% of total cell count, 21 to 112 cells) MLR. This suggests that the MLR sends similar axonal projections to RS cells on both sides, in accord with our physiological results.

### Comparison of Ca^2+^ responses in the mRN and the iRN

**Figure 7:**
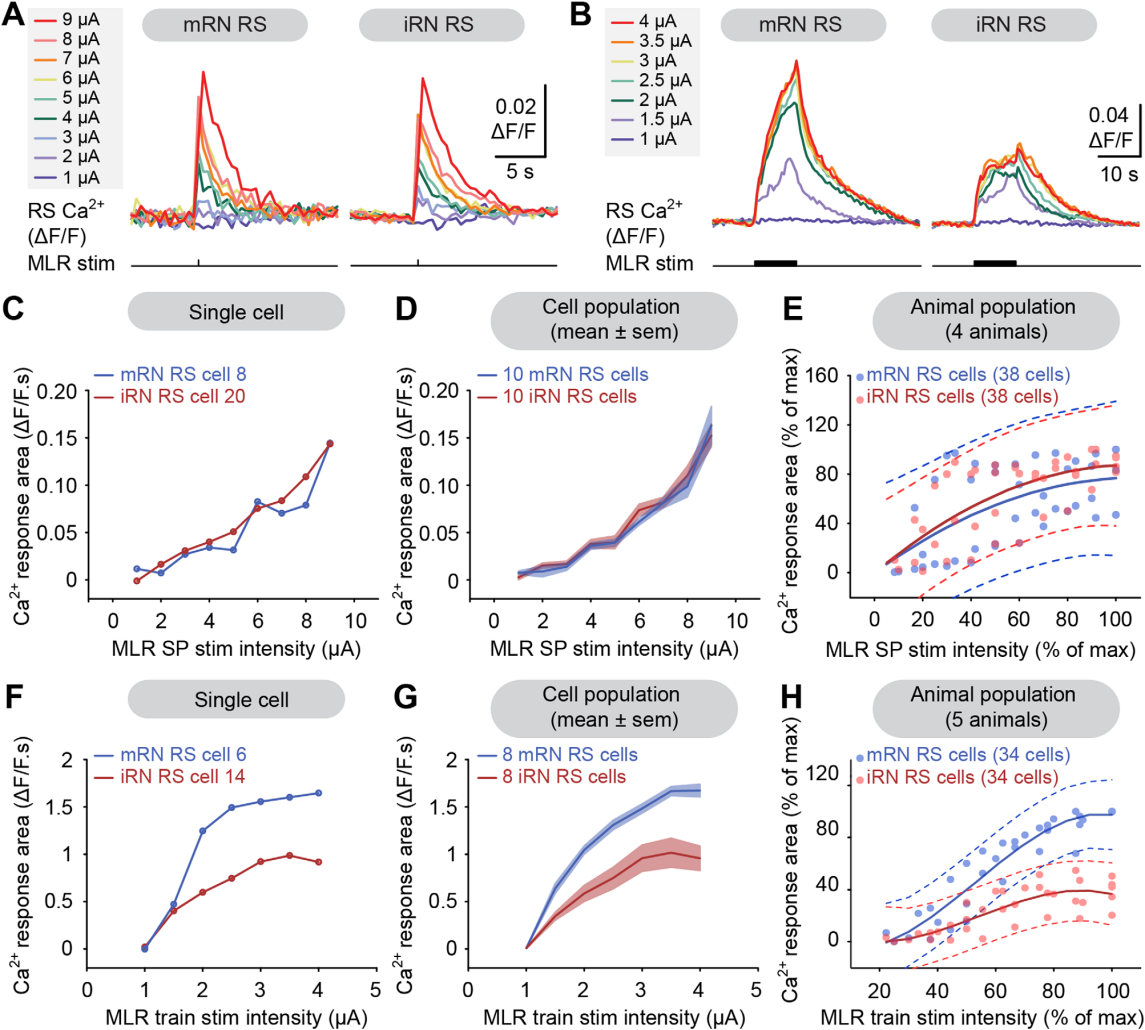
Comparison of Ca^2+^ responses in the middle (mRN) and inferior reticular nuclei (iRN). **A**: Ca^2+^ responses (ΔF/F) in individual RS cells in the mRN and iRN in response to single pulse (SP) MLR stimulation (1–9 μA, 2 ms pulses). **B**: Ca^2+^ responses (ΔF/F) in individual RS cells in the mRN and iRN in response to trains of stimulation applied to the MLR (1–4 μA, 10 seconds train, 5 Hz, 2 ms pulses). **C**: Plot illustrating the size of Ca^2+^ responses in two RS cells (mRN: blue trace; iRN: red trace) vs. the MLR SP stimulation intensity. The corresponding raw data for these cells are illustrated in (A). **D**: Same as in (C), but after compiling the responses (mean ± SEM) of 10 RS cells in the mRN (blue) and in the iRN (red). **E**: Relationships between the size of the Ca^2+^ responses of mRN (blue dots, *n* = 38 cells) and iRN (red dots, *n* = 38 cells) RS cells pooled from four preparations and the intensity of MLR SP stimulation (1–12 μA, 2 ms pulses). The mRN data (R^2^ = 0.33 *P* < 0.01) and iRN data (R^2^ = 0.51 *P* < 0.001) followed a cubic polynomial function (blue and red solid lines, respectively). The dotted lines illustrate the 95% prediction intervals for each function. **F**: Plot illustrating the size of Ca^2+^ responses in two RS cells (mRN: blue trace; iRN: red trace) vs. the MLR train stimulation intensity. The corresponding raw data for these cells are illustrated in (B). **G**: Same as in (C), but after compiling the responses (mean ± SEM) of eight RS cells in the mRN (blue) and in the iRN (red). **H**: Relationships between the normalized Ca^2+^ responses of mRN (blue dots, *n* = 34 cells) and iRN (red dots, *n* = 34 cells) RS cells pooled from five preparations and the intensity of trains of MLR stimulation (1–5 μA, 10 seconds train, 5 Hz, 2 ms pulses). The mRN data (R^2^ = 0.89, *P* < 0.001) and iRN data (R^2^ = 0.63, *P* < 0.001) followed a cubic polynomial function (blue and red solid lines, respectively). The dotted lines illustrate the 95% prediction intervals for each function. Data from A,C,D and B,F,G are from two different preparations.

To examine whether the RS neurons of the rostral and caudal hindbrain were recruited in a similar manner, Ca^2+^ responses were recorded simultaneously in RS cells of the mRN and iRN. Single pulse stimulation in the MLR elicited Ca^2+^ responses in both nuclei (Fig. 7A,C,D). The size of the responses increased in a similar fashion in the two nuclei as the stimulation strength was increased (*n* = 38 mRN cells and *n* = 38 iRN cells pooled from four preparations, Fig. 7E). The maximal responses were of comparable size. Indeed, 95% (40 of 42 values) of mRN responses were located in the prediction interval of iRN cells, whereas 100% (42 of 42 values) of iRN responses were located below the prediction interval of mRN cells. On the other hand, when trains of stimuli (1–5 μA, 10 seconds train, 5 Hz, 2 ms pulses) were used, the maximal responses (Fig. 7B,F,G) were larger in RS cells of the mRN compared to the iRN (34 mRN and 34 iRN cells pooled from five preparations). The difference in maximal amplitude was confirmed by calculating the 95% prediction intervals for pooled data (Fig. 7H). Indeed, 69% (24 of 35 values) of the mRN responses were above the prediction interval for iRN RS cells. Conversely, 63% (22 of 35 values) of iRN responses were below the prediction interval of mRN RS cells. This suggests that Ca^2+^ responses in iRN cells “saturate” at lower amplitude than mRN cells. On the other hand, the recruitment pattern for the two groups of RS cells was similar as the MLR stimulation intensity was increased.

### Glutamatergic transmission from the MLR to RS cells

Next we examined the neurotransmitters involved in the activation of RS cells by the MLR. In lampreys, the MLR was shown to send glutamatergic (Brocard and Dubuc, 2003; Le Ray et al., 2003; see also Villar‐Cervino et al., 2013) and cholinergic (Le Ray et al., 2003) inputs to RS neurons. Here, we focused on the role of glutamatergic neurotransmission in salamanders.

**Figure 8:**
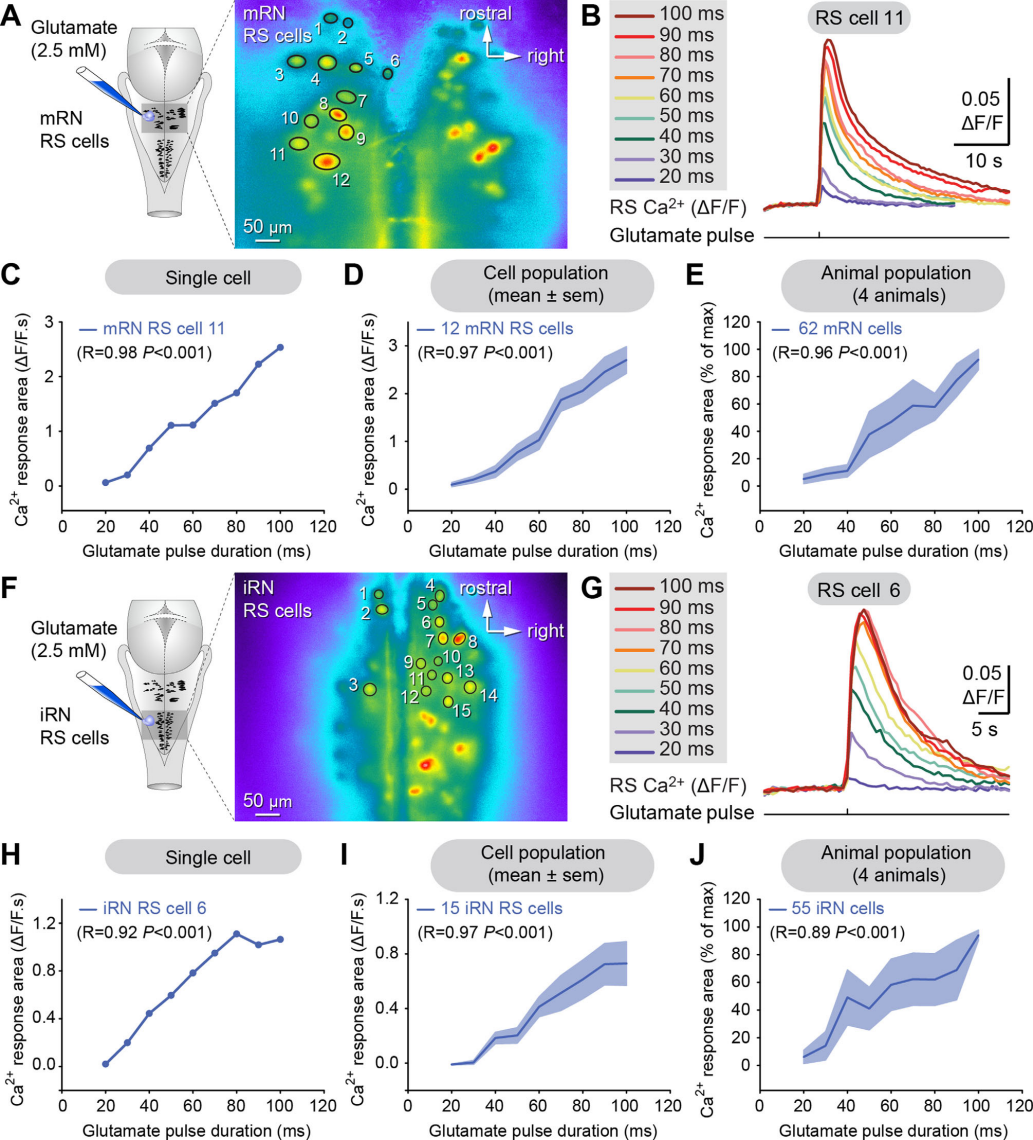
Glutamate elicits graded Ca^2+^ responses in reticulospinal (RS) neurons of the middle (mRN) and inferior reticular nuclei (iRN). **A**: Left: Schematic dorsal view of the salamander brain. Right: Ca^2+^ fluorescence at rest of RS cells of the mRN. **B**: Ca^2+^ responses (ΔF/F) in individual RS cell of the mRN in response to local microinjections of 0.09 to 3.04 pmol of D,L‐glutamate (2.5 mM, 20–100 ms pulse duration, 3–4 PSI, pipette diameter 10–20 μm, 0.04–1.21 nL per microinjection). **C**: Plot illustrating the size of Ca^2+^ responses in one RS cell in the mRN vs. the glutamate pulse duration. The corresponding raw data for this cell are illustrated in (B). **D**: Same as in (C), but after compiling the responses (mean ± SEM) of 12 RS cells of the mRN. **E**: Plot illustrating the normalized Ca^2+^ responses (mean ± SEM) of a pool of mRN RS cells (*n* = 62 cells from four preparations) as a function of the glutamate pulse duration. **F**: Left: Schematic dorsal view of the salamander brain. Right: Ca^2+^ fluorescence at rest of RS cells of the iRN. **G**: Ca^2+^ responses (ΔF/F) in individual RS cell of the iRN in response to local microinjections of 0.09 to 3.04 pmol of D,L‐glutamate (2.5 mM, 20–100 ms pulse duration, 3–4 PSI, pipette diameter 10–20 μm, 0.04–1.21 nL per microinjection). **H**: Plot illustrating the size of Ca^2+^ responses in one RS cell in the iRN vs. the glutamate pulse duration. The corresponding raw data for this cell are illustrated in (G). **I**: Same as in (H), but after compiling the responses (mean ± SEM) of 15 RS cells of the iRN. **J**: Plot illustrating the normalized Ca^2+^ responses (mean ± SEM) of a pool of iRN RS cells (*n* = 55 cells from four preparations) as a function of the glutamate pulse duration. In C–E and H–J, the coefficient of correlation (R) and the corresponding significance (*P*) are indicated. A–E, F–J, data are from two different preparations.

We first examined the responses of RS neurons to local pressure ejection of glutamate (2.5 mM, 20–100 ms pulse duration, 0.04–1.21 nL per microinjection). The ejection of 0.09 to 3.04 pmol of D,L‐glutamate on RS neurons elicited clear Ca^2+^ responses in RS neurons both in the mRN (Fig. 8A–D) and the iRN nuclei (Fig. 8F–I). Increasing the duration of the glutamate pulse (i.e., the ejected amount of glutamate) produced a linear increase in the responses in the mRN (R = 0.96, *P* < 0.001, *n* = 62 cells pooled from four preparations, Fig. 8E) and in the iRN (R = 0.89, *P* < 0.001, *n* = 55 cells pooled from four preparations, Fig. 8J). In two control experiments, glutamate was replaced with Ringer's and no Ca^2+^ response was observed, confirming that the RS cells responded to glutamate.

**Figure 9:**
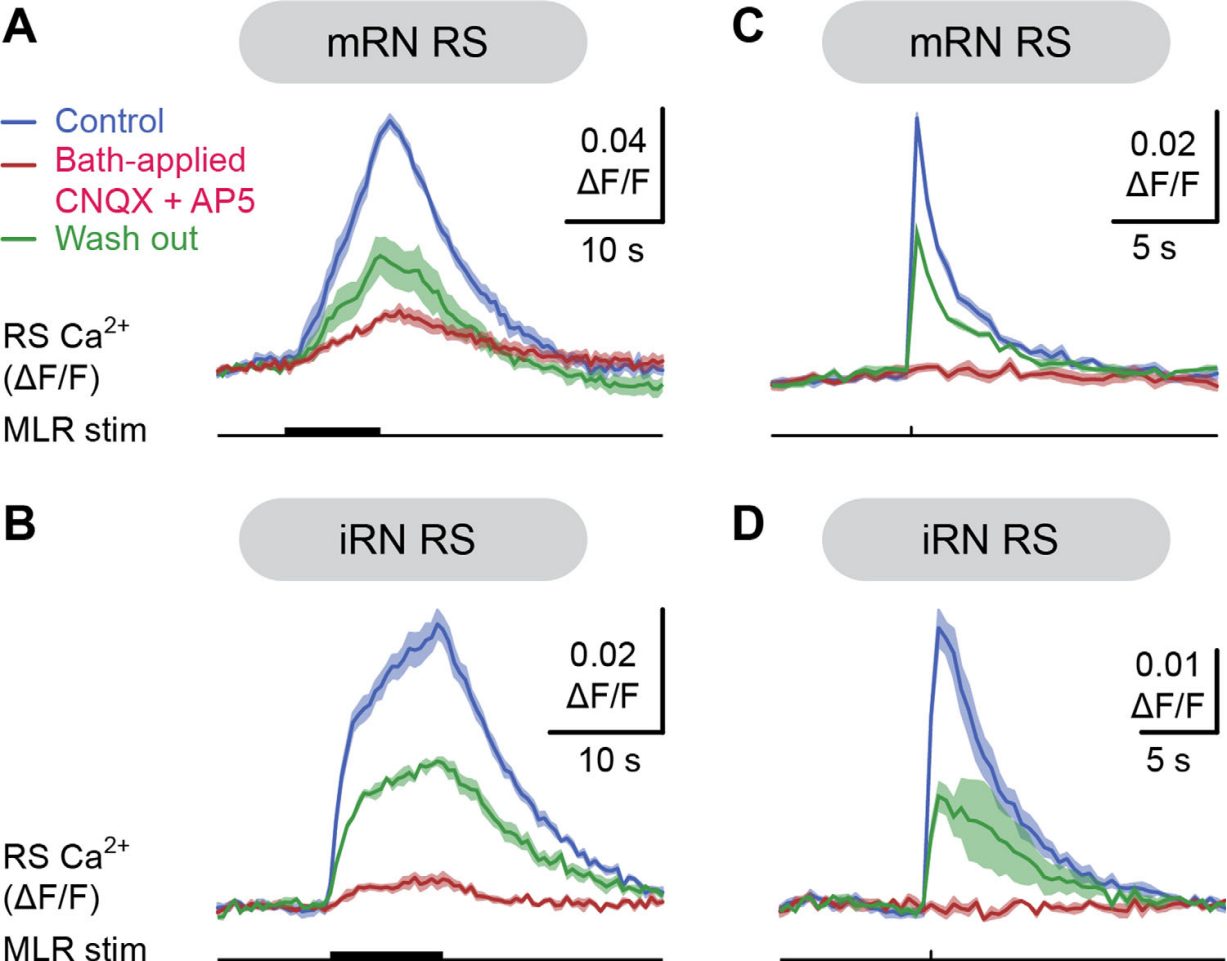
Effect of bath‐application of glutamatergic antagonists on the Ca^2+^ responses of reticulospinal (RS) cells evoked by MLR stimulation. **A,B**: Effect of the bath application of the glutamatergic antagonists CNQX (25 μM) and AP5 (100 μM) on the Ca^2+^ responses (ΔF/F) of individual RS cells of the mRN (A) and iRN (B) in response to trains of MLR stimulation (mRN cell, 5 μA, 10 seconds train, 5 Hz, 2 ms pulses; iRN cell, 2.5 μA, 10 seconds train, 5 Hz, 2 ms pulses). For each of the three conditions (control, drug application, washout), data are illustrated as mean ± SEM, with five trials per condition for the mRN cell, and four trials per condition for the iRN cell. Both for the mRN and iRN data, the glutamatergic antagonists were bath‐applied during 7 minutes. The effects decreased 60 minutes after washout for the mRN, and 80 minutes after washout for the iRN. **C,D**: Effect of the bath application of the glutamatergic antagonists CNQX (25 μM) and AP5 (100 μM) on the Ca^2+^ responses (ΔF/F) of individual RS cells of the mRN (C) and iRN (D) in response to MLR single pulse stimulation (mRN data, 5 μA, 2 ms pulses; iRN data, 9 μA, 2 ms pulses). For each of the three conditions (control, drug application, washout), data are illustrated as mean ± SEM, with five trials per condition for the mRN cell, and four trials per condition for the iRN cell. The glutamatergic antagonists were bath‐applied during 6 minutes for the mRN cell, and during 7 minutes for the iRN cell. The effects decreased 90 minutes after washout for the mRN, and 80 minutes after washout for the iRN. A–D, data are from four different preparations.

We next examined whether glutamatergic neurotransmission was involved in generating RS responses to MLR stimulation. When glutamatergic antagonists targeting AMPA/kainate receptors (CNQX, 25 μM) and NMDA receptors (AP5, 100 μM) were added to the bathing Ringer's solution, the Ca^2+^ responses were dramatically reduced in RS cells of the mRN (Fig. 9A) and the iRN (Fig. 9B). The responses in the mRN were reduced to 10.3 ± 1.0% of control (*P* < 0.05 vs. control, Student‐Newman‐Keuls test after a Friedman repeated‐measures ANOVA on ranks *P* < 0.001, *n* = 56 cells pooled from four preparations). They increased back to 26.0 ± 3.1% of control (*P* < 0.05 vs. CNQX/AP5, Student‐Newman‐Keuls test, *n* = 56 cells pooled from four preparations) after a 60–80‐minute washout of the glutamatergic antagonists. In the iRN, the responses were reduced to 1.2 ± 4.0% of control after bath‐application of the antagonists (*P* < 0.05 vs. control, Student‐Newman‐Keuls test after a Friedman repeated‐measures ANOVA on ranks *P* < 0.001, *n* = 32 cells pooled from three preparations) and increased back to 48.4 ± 7.6% of control (*P* < 0.05 vs. CNQX/AP5, Student‐Newman‐Keuls test) after a 60–90‐minute washout.

The effects of the glutamatergic antagonists were tested on Ca^2+^ responses evoked by MLR stimulation comprising only single pulses to reduce the recruitment of polysynaptic inputs. Indeed, it was previously proposed that synaptic responses elicited in salamander RS cells comprised large polysynaptic components when trains of stimuli were used (Bar‐Gad et al., 1999; Kagan and Shik, 2004). The Ca^2+^ responses induced in RS cells of the mRN to single pulse MLR stimulation (5–10 μA, 2 ms pulses, Fig. 9C) were markedly reduced to 8.1 ± 4.0% of control (*P* < 0.001 vs. control, Student‐Newman‐Keuls test after a one‐way ANOVA *P* < 0.001, *n* = 24 cells pooled from two preparations) after bath application of CNQX and AP5. The responses increased back to 35.4 ± 5.9% of control (*P* < 0.001 vs. CNQX/AP5, Student‐Newman‐Keuls test) after washing out the antagonists for 90 to 120 minutes. Similar effects were observed in RS cells of the iRN (9–10 μA, 2 ms pulses, Fig. 9D). The Ca^2+^ responses decreased to 1.2 ± 0.9% of control following bath application of CNQX and AP5 (*P* < 0.05 vs. control, Student‐Newman‐Keuls test after a Friedman ANOVA on ranks for repeated measures *P* < 0.001, *n* = 24 cells pooled from two preparations). They recovered to 47.0 ± 6.5% of control after washout for 90 to 180 minutes (*P* < 0.05 vs. CNQX/AP5, Student‐Newman‐Keuls test).

**Figure 10:**
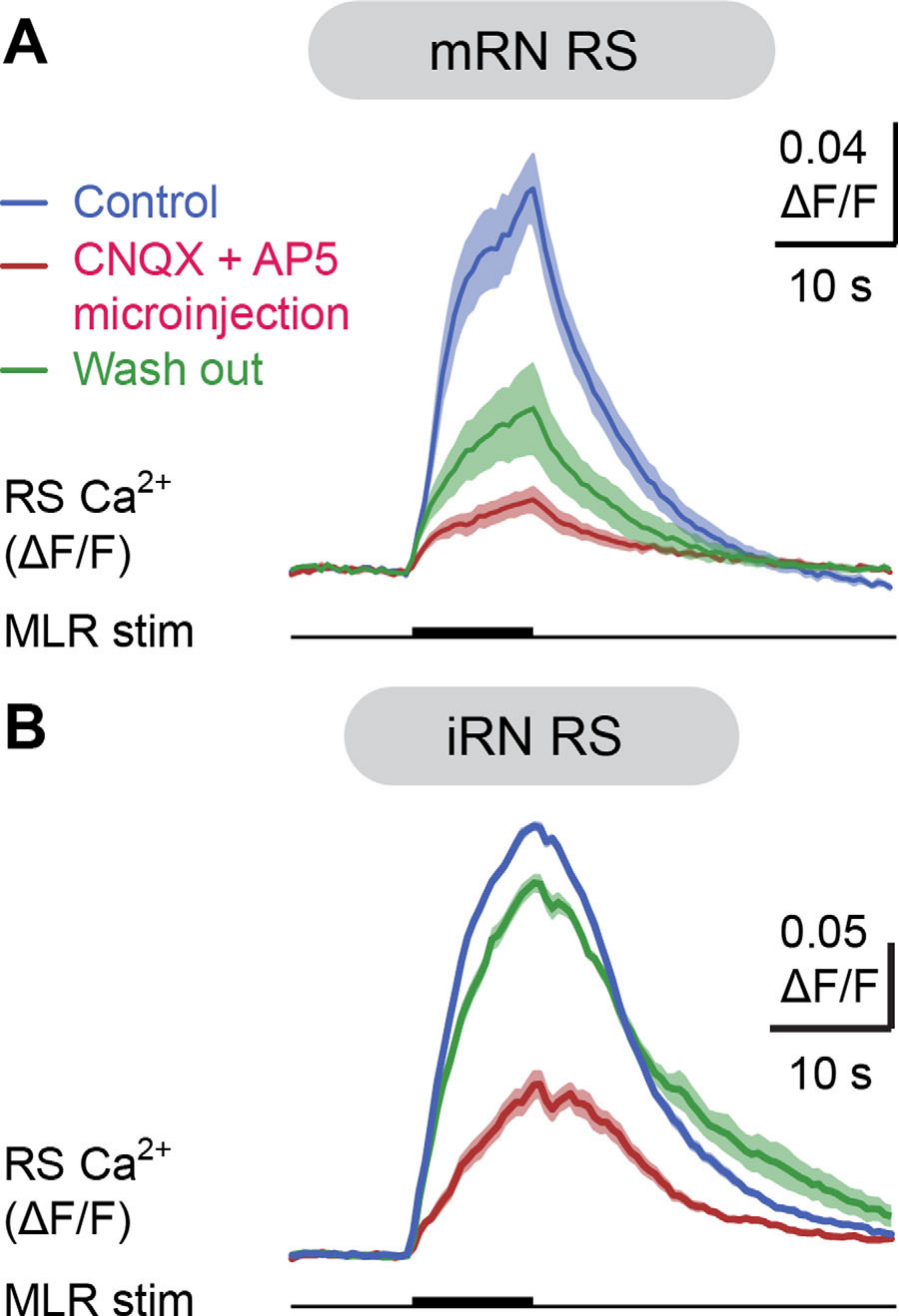
Local microinjections of glutamatergic antagonists onto the middle (mRN) or inferior reticular nucleus (iRN) abolish their Ca^2+^ responses evoked by MLR stimulation. **A,B**: Comparison of the Ca^2+^ responses (ΔF/F) of individual RS cells of the mRN (A) and iRN (B) in response to trains of MLR stimulation (mRN cell: 8 μA, 10 seconds train, 5 Hz, 2 ms pulses; iRN cell: 4 μA, 10 seconds train, 5 Hz, 2 ms pulses) before and after local microinjection onto the mRN (A) or iRN (B) of a solution containing the 3.6 pmol of CNQX and 1.8 pmol of AP5 (CNQX 1 mM; AP5 500 μM; 20 pulses, 3–4 PSI, 40 ms pulses, 2 Hz, 3.6 nL per microinjection). For each of the three conditions (control, drug application, washout), data is illustrated as mean ± SEM, with five trials per condition both for the mRN cell and the iRN cell. The effects decreased 90 minutes after washout for the mRN cell, and 135 minutes after washout for the iRN cell. A,B, data are from two different preparations.

We then tested whether similar effects were observed when locally blocking glutamatergic neurotransmission at the level of RS neurons. Following microinjections of 3.6 pmol of CNQX (1 mM) and 1.8 pmol of AP5 (500 μM) over the mRN, the Ca^2+^ responses in RS cells in this nucleus (Fig. 10A) decreased to 21.9 ± 3.5% of control (*P* < 0.05 vs. control, Student‐Newman‐Keuls test after a Friedman repeated‐measures ANOVA on ranks *P* < 0.001, *n* = 60 cells pooled from four preparations). The responses grew back to 50.2 ± 5.3% of control (*P* < 0.05 vs. CNQX/AP5, Student‐Newman‐Keuls test) after a 90–135‐minute washout of the antagonists. Similarly, a local application of 3.6 pmol of CNQX (1 mM) and 1.8 pmol of AP5 (500 μM) over the iRN (Fig. 10B) reduced the Ca^2+^ responses elicited by trains of MLR stimulation (3–6 μA, 2 ms pulses, 5 Hz, 10 seconds train) to 30.4 ± 0.5% of control (*P* < 0.05 vs. control, Student‐Newman‐Keuls test after a Friedman ANOVA on ranks for repeated measures *P* < 0.001, *n* = 57 cells pooled from four preparations). These responses recovered to 69.4 ± 0.9% of control (*P* < 0.05 vs. CNQX/AP5, Student‐Newman‐Keuls test) after a 60–90‐minutes washout of the antagonists. Altogether, these findings indicate that glutamatergic neurotransmission at the level of RS cells contributes markedly to the excitation from the MLR to RS neurons in the two reticular nuclei.

### Glutamatergic cells in the MLR

**Figure 11:**
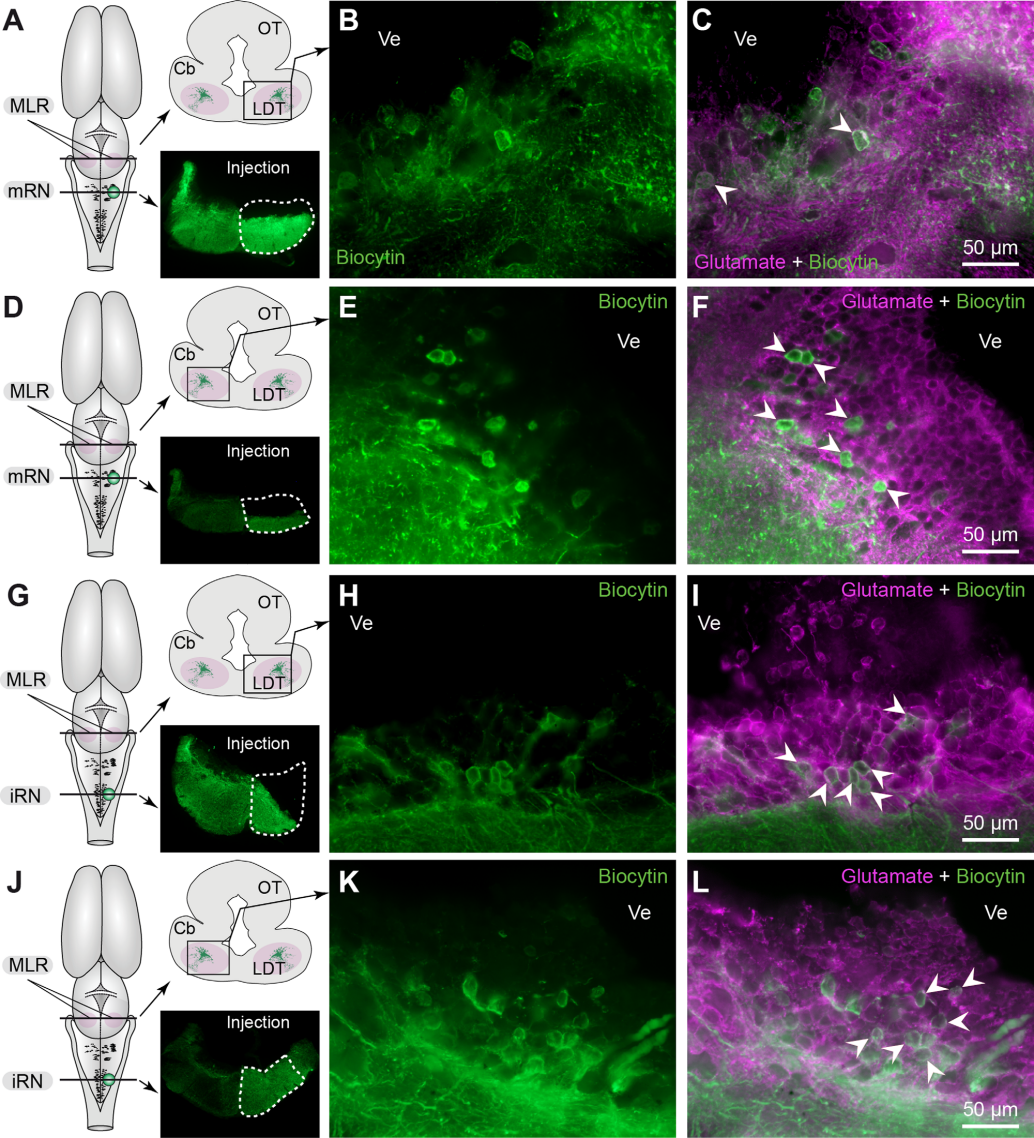
Glutamate‐immunopositive cells in the MLR send descending projections on both sides in the middle (mRN) and inferior (iRN) reticular nuclei. **A**: Schematic dorsal view of the salamander brain illustrating the approximate location of the MLR cross‐section shown in (B,C). On the schematized brain section, the MLR is delineated in pink. The photomicrograph illustrates the tracer (biocytin, green) injection site in the mRN. The site is enclosed by a white dashed line. **B**: Retrogradely labeled neurons in the MLR ipsilateral to the injection site. **C**: Retrogradely labeled neurons in the MLR ipsilateral to the injection site showing immunoreactivity against glutamate (white arrowheads). **D–F**: Same representation as in (A–C). Retrogradely labeled neurons in the MLR contralateral to the injection site in the mRN showing immunoreactivity against glutamate (white arrowheads). **G–I**: Same representation as in (A–C). Retrogradely labeled neurons in the MLR ipsilateral to the injection site in the iRN showing immunoreactivity against glutamate (white arrowheads). **J–L**: Same representation as in (A–C). Retrogradely labeled neurons in the MLR contralateral to the injection site in the iRN showing immunoreactivity against glutamate (white arrowheads). Cb, cerebellum; LDT, laterodorsal tegmental nucleus; OT, optic tectum; Ve, ventricule.

Anatomical experiments combining retrograde tracing and immunofluorescence were carried out to determine whether some of the MLR cells projecting to the reticular formation were glutamatergic. Biocytin was injected in either the mRN or the iRN and immunofluorescence against glutamate was performed. Several glutamate‐positive neurons were retrogradely labeled in the MLR both ipsilaterally (Fig. 11A–C) and contralaterally (Fig. 11D–F) to the tracer injection in the mRN (three preparations). Similarly, glutamate‐positive neurons were found in three preparations on both sides of the MLR following retrograde labeling from the iRN (ipsilaterally to tracer injection, see Fig. 11G–I, contralaterally see Fig. 11J–L). Many of these cells were located in the LDT where a large population of cholinergic neurons was found by us and others (Marin et al., 1997; Cabelguen et al., 2003) (see Fig. 1B,C).

## DISCUSSION

Our study shows that the MLR sends descending inputs to hindbrain RS cells of the mRN and iRN. The inputs are strikingly similar on both sides and are very powerful because single pulses of MLR stimulation elicited significant responses in all of the recorded RS cells. Bath‐applications of glutamatergic blockers markedly reduced the responses as well as local microinjections of glutamatergic antagonists over the RS cells, indicating that glutamate neurotransmission plays a crucial role. In addition, glutamate‐immunoreactive cells projecting down to RS cells were found in the MLR. Altogether, our study provides a detailed anatomical and physiological account of the MLR projections to RS cells in a tetrapod. This brainstem circuit is likely to play a major role in the control of locomotion in salamanders as previously shown or proposed for other vertebrate species.

### Central role of the RS system in locomotor control

A thorough understanding of the supraspinal control of locomotion requires a detailed characterization of the anatomy and physiology of MLR projections to RS cells. The RS system was previously described as the “final common descending pathway” for locomotion (for review, see Dubuc et al., 2008). It plays a crucial role in activating the spinal locomotor networks. The pioneer work of the Russians in cats not only provided the discovery of the MLR (Shik et al., 1966), but also the first evidence that RS cells relay MLR inputs to the spinal locomotor networks (Orlovsky, 1970). This was later confirmed anatomically and physiologically by others in cats and in other species (cats: Steeves and Jordan, 1980; Garcia‐Rill and Skinner, 1987a, 1987b; Noga et al., 1988, 1991; birds: Sholomenko et al., 1991). In cats, RS cells were shown to be active during locomotion on a treadmill (Orlovsky, 1970; for review see Shik and Orlovsky, 1976; Drew et al., 1986), MLR‐evoked locomotion (Iwakiri et al., 1995), and fictive locomotion (Perreault et al., 1993).

Studies in lampreys have provided far more detailed information on the role of RS cells in the control of locomotion. RS cells were found to provide the descending glutamatergic excitation to the spinal locomotor networks in lampreys (Buchanan and Grillner, 1987; Brodin et al., 1988; Ohta and Grillner, 1989; Brocard and Dubuc, 2003) and receive direct glutamatergic (Brocard and Dubuc, 2003; Le Ray et al., 2003) and cholinergic (Le Ray et al., 2003) inputs from the MLR. They also receive an indirect MLR input through the activation of a group of brainstem muscarinoceptive cells that in turn send an additional excitation to RS cells (Smetana et al., 2010). In salamanders, the spinal locomotor networks can also be activated by pharmacologically mimicking the descending RS drive with glutamatergic agonists (Wheatley et al., 1994; Cheng et al., 1998; Delvolvé et al., 1999; Ryczko et al., 2010, 2015; Charrier and Cabelguen, 2013). The anatomy of the RS system was described in this animal by tracing studies (Naujoks‐Manteuffel and Manteuffel, 1988; Sanchez‐Camacho et al., 2001; Hubbard et al., 2010) and some RS cells were shown to be glutamatergic (Chevallier et al., 2004). The key role of RS cells in controlling the spinal locomotor CPG was recently modeled (Bicanski et al., 2013). The salamander MLR controls locomotor output (Cabelguen et al., 2003), and we now show that the strength of MLR stimulation controls the Ca^2+^ responses of RS neurons of the rostral and caudal hindbrain. These responses are consistent with those observed with extracellular recordings of the reticular formation following MLR stimulation (Bar‐Gad et al., 1999; Kagan and Shik, 2004) or during locomotion in salamanders (Lowry et al., 1996; Hubbard et al., 2010).

In mice, optogenetic activation of glutamatergic neurons in the caudal hindbrain evokes fictive locomotion (Hägglund et al., 2010). The activated glutamatergic cells are likely functionally similar to the lamprey RS cells and the glutamatergic cells in the zebrafish hindbrain that are active during locomotion (Kinkhabwala et al., 2011). The activation of the latter cells was shown to be necessary and sufficient to elicit locomotion (Kimura et al., 2013). How the RS system is controlled by the MLR in mammals remains unresolved. In mice, around 20% of glutamatergic RS cells receive inputs from the MLR and show increased c‐fos expression after the animals are made to walk (Bretzner and Brownstone, 2013). It is possible that these cells receive glutamatergic inputs from the MLR, because the optogenetic activation of glutamatergic neurons in the MLR evokes locomotion (Lee et al., 2014). Uncovering the mechanisms by which the MLR controls RS cells is also important because deep brain stimulation of the MLR improves locomotor function in patients suffering from Parkinson's disease (Mazzone et al., 2005; Plaha and Gill, 2005). Moreover, MLR deep brain stimulation promotes locomotor recovery in rodents with partial spinal cord injury (Bachmann et al., 2013).

### Descending inputs from the MLR

Stimulation of the MLR on one side was shown to generate bilaterally symmetrical locomotor movements in the cat (Shik et al., 1966; Musienko et al., 2012), lampreys (Sirota et al., 2000; Brocard et al., 2010), stingrays (Bernau et al., 1991), rabbits (Musienko et al., 2008), and guinea‐pigs (Marlinksy and Voitenko, 1991). In lampreys, it was proposed that this resulted at least in part from bilaterally symmetrical inputs from the MLR to RS cells on the basis of anatomical tracing, intracellular recordings, and Ca^2+^ imaging of RS cells (Brocard et al., 2010). Orlovsky (1970) provided the first evidence in a tetrapod that RS cells receive inputs from the MLR on both sides but did not determine whether these inputs were bilaterally similar because cells were not simultaneously recorded on both sides. We now provide anatomical and physiological evidence in salamanders that the MLR sends bilaterally similar inputs to RS cells of the rostral and caudal hindbrain. This is consistent with the bilaterally symmetrical locomotor output recorded in salamanders upon MLR stimulation on one side (Cabelguen et al., 2003). Double intracellular recordings are needed to confirm that RS firing and depolarization on both sides are similar following unilateral MLR stimulation, as previously shown in lampreys (Brocard et al., 2010). Finally, it would be interesting to determine in the future whether a single MLR cell projects both ipsi‐ and contralaterally, as previously examined in lampreys by injecting a retrograde tracer on each side (Brocard et al., 2010).

A striking finding of the present study was the powerful inputs from the MLR to RS cells on both sides. The RS cells displayed sharp onset responses even when using single pulses of stimulation in the MLR. Responses were seen using stimulation intensities as low as 2 μA. However, our Ca^2+^ imaging approach is too slow (2 Hz sampling) to conclude on the mono‐ or polysynaptic nature of the MLR‐RS connection. On the other hand, based on extracellular recordings of unidentified hindbrain neurons, it was proposed that the MLR activated monosynaptically only a small part of the recorded cells (Bar‐Gad et al., 1999; Kagan and Shik, 2004). Our anatomical experiments show that the MLR sends projections to both the mRN and the iRN. It is possible that these projections make monosynaptic connections onto RS cells, but single cell electrophysiology would be needed to confirm this. Monosynaptic connections (MLR‐RS) were also shown in lampreys (Brocard and Dubuc, 2003; Brocard et al., 2010). In cats, extracellular data strongly suggested that cells in the reticular formation receive monosynaptic inputs from the MLR (Orlovsky, 1970; Iwakiri et al., 1995). In accord with this, monosynaptic connections were also recently proposed in rodents (Bretzner and Brownstone, 2013). Our results suggest that the MLR may directly control the activity of the RS cells that in turn activate the spinal locomotor networks. Our anatomical results revealed very symmetrical projections from the MLR to RS cells, even more so than reported previously in lampreys (Brocard et al., 2010) and mammals (Steeves and Jordan, 1984; Garcia‐Rill et al., 1986; Bachmann et al., 2013).

### MLR sends a major glutamatergic drive to RS cells

We have shown that large Ca^2+^ responses were elicited by local application of glutamate directly over RS cells. In addition, MLR‐induced Ca^2+^ responses were nearly abolished in the presence of glutamate receptor antagonists. Local applications of glutamatergic antagonists were slightly less effective than bath applications. This is most probably due to the inherent difficulty of blocking all receptors with local microinjection because the diffusion of the antagonists cannot be perfectly controlled. Using immunofluorescence coupled with neural tracing, we found glutamate neurons in the MLR that project down to the rhombencephalic reticular formation, further supporting that the MLR sends glutamatergic inputs to RS neurons in salamanders. We are aware that glutamate immunostaining is not a definitive proof for a glutamatergic identity of a neuron, as glutamate is known to be the metabolic precursor of GABA. Colocalization of the glutamate and GABA was reported in the hindbrain of frogs (Reichenberger et al., 1997) and lampreys (Villar‐Cervino et al., 2011). It was also seen in the visual system of salamanders (Yang, 1996), cats (Jojich and Pourcho, 1996), and humans (Davanger et al., 1991). In the present study both physiological and anatomical findings concur to support the presence of descending MLR glutamatergic projections to RS neurons as shown in lampreys (Brocard and Dubuc, 2003; Le Ray et al., 2003). Glutamatergic inputs are known to play a central role in the initiation and the maintenance of locomotor movements in lampreys. Local injections of glutamatergic antagonists over RS cells of the pons (middle rhombencephalic reticular nucleus “MRRN” in lampreys; mRN in the present study) dramatically increased the amount of current needed to elicit locomotion with MLR stimulation (Brocard and Dubuc, 2003). The glutamate antagonists also greatly decreased the frequency of swimming and the amplitude of EMG bursts. When glutamatergic antagonists were injected over RS cells of the caudal hindbrain (posterior rhombencephalic reticular nucleus “PRRN” in lampreys, iRN in the present study), swimming frequencies decreased, but swimming initiation was not prevented (Brocard and Dubuc, 2003). In line with this, optogenetic stimulation of glutamatergic cells in the MLR elicits locomotion in vivo in mice (Lee et al., 2014). Altogether, our results along with those of others strongly suggest that, in salamanders, the glutamatergic inputs from the MLR to RS cells are very likely responsible for the initiation and the control of the frequency of locomotor movements exerted by MLR stimulation (Cabelguen et al., 2003).

The remaining component of the RS responses that we observe in some cases after blocking glutamatergic transmission is possibly cholinergic. In salamanders, efficient MLR stimulation sites (Cabelguen et al., 2003) are located within cholinergic cells in the LDT (Marin et al., 1997). We now confirm this observation. The location of cholinergic cells has been considered an important anatomical landmark for the MLR in other species (lampreys: Le Ray et al., 2003; rats: Garcia‐Rill et al., 1987; monkeys and humans: Karachi et al., 2010). In lampreys, there is a significant cholinergic component to the MLR inputs to RS cells (Le Ray et al., 2003). In monkeys and humans, degeneration of MLR cholinergic cells is associated with locomotor deficits (Karachi et al., 2010). The role of the MLR cholinergic cells in the activation of RS cells and in locomotor control remains to be characterized in salamanders.

The MLR was defined functionally almost 50 years ago in cats by Shik et al. (1966). Although the name of the region presumes that it is located in the mesencephalon, it is known that, in some species, part of the MLR is located in the rhombencephalon (e.g., lampreys: Le Ray et al., 2003; Brocard et al., 2010; for review, see Ryczko and Dubuc, 2013; rats: Garcia‐Rill et al., 1987). In the present study, labeled MLR cells were located in rhombomere 0 (r0, isthmus) and rhombomere 1 (r1) territories previously described in salamanders (Morona and Gonzales, 2009), lampreys (Murakami et al., 2004), and mice (Moreno‐Bravo et al., 2014; for review, see Puelles et al., 2013). On the basis of the patterns of genetic expression during embryogenesis, these territories comprise the PPN and the LDT in amphibians (e.g., Morona and Gonzalez, 2009) and mammals (e.g., Waite et al., 2012; Moreno‐Bravo et al., 2014). Because these structures are considered to be part of the MLR (for review, see Ryczko and Dubuc, 2013), the latter can be considered to be partly located in the rhombencephalon.

### Recruitment of RS cells by MLR stimulation

In the present study we did not see a sequential recruitment of RS cells in the mRN and iRN as the MLR stimulation strength was increased. Responses were observed in the two nuclei at the same minimal stimulation intensity. However, the maximal Ca^2+^ responses in iRN cells were smaller than in the mRN. The differential responses were seen only with trains of stimuli and not with single pulses. In addition to possible monosynaptic inputs, repetitive MLR stimulation could recruit some local excitatory network that would be more powerful at the level of the mRN. Polysynaptic inputs were previously proposed to be present in the MLR‐RS circuit (Bar‐Gad et al., 1999; Kagan and Shik, 2004). Differences in the intrinsic properties of the RS cells of the mRN and iRN cannot be excluded. In zebrafish, different reticular cells are recruited when locomotor speed increases: the dorsal glutamatergic neurons are recruited first, at low swimming frequencies (Kinkhabwala et al., 2011). In lampreys, RS cells in the caudal hindbrain (PRRN) display smaller MLR‐evoked responses and begin spiking at higher MLR stimulation strengths than rostral hindbrain (MRRN) RS cells (Brocard and Dubuc, 2003) and therefore the MLR inputs to the two groups of RS cells are not the same as we now show for salamanders.

Intracellular recordings of paired RS neurons on each side of the brainstem in the mRN and iRN are needed to determine the relationship between neuron firing and the calcium responses. It is not possible to predict with 100% accuracy the firing of each neuron on the basis of their calcium responses alone. However, simultaneous calcium imaging (also with calcium green dextran amine) and electrophysiological recordings of the same cells were performed in zebrafish (Bhatt et al., 2007; Wang and McLean, 2014). It was shown that a 9% increase in ΔF/F was associated with cell firing. When applied to our results, this criterion would indicate that trains of stimulation induce suprathreshold activity in most neurons of the mRN and iRN (see the ΔF/F scale values in Figs. 2B,C and 4B,C, respectively). This is consistent with the spinal cord recordings of the descending RS fibers, showing that spiking activity increases with the intensity of MLR stimulation (Figs. 2C and 4C). It is likely that the absence of spinal cord in our preparations will reduce the excitability of the RS cells. Indeed, it was shown in lampreys that rhythmic modulation of RS cells entirely results from spinal inputs (Einum and Buchanan, 2004, 2005, 2006; Antri et al., 2009).

Whether RS cells of the mRN and iRN play a specific role in the transition from stepping to swimming remains also to be determined in salamanders. In a previous modeling study, the absence of limb movements during swimming (Frolich and Biewener, 1992; Delvolvé et al., 1997; Ryczko et al., 2015) was proposed to result from “saturation” of limb networks for high MLR stimulation (Ijspeert et al., 2007). The saturation was proposed to be due either to a limited capability of limb networks to produce rhythmic output at high frequency and/or a mechanism by which the descending command (RS activity) to limb networks would stop when MLR drive exceeded a threshold. In the present study, none of the RS cells that reached a maximal response displayed any decrease in activity when increasing further the MLR stimulation intensity. On the other hand, we obviously did not record from all RS cells. Despite the strength of the Ca^2+^ imaging approach over intracellular recordings, it still provides only a sampling of the cell population under study. A spinal mechanism underlying the switching between stepping and swimming may also be at play. For instance, spinal modules of interneurons and motoneurons were recently described in zebrafish (McLean et al., 2007; Ampatzis et al., 2014) and mice (Talpalar et al., 2013) and their activity was proposed to account for changes in locomotor speed and pattern. It will be of interest to eventually characterize the spinal targets of the two groups of RS cells in salamanders.

## CONCLUSION

The MLR‐RS circuit of salamanders shows striking similarities with that previously described at the cellular level in lampreys (Brocard and Dubuc, 2003; Le Ray et al., 2003; Brocard et al., 2010). Because the salamander is regarded as the tetrapod most closely resembling the first terrestrial vertebrates (Gao and Shubin, 2001), it is likely that the organization of brainstem locomotor circuits was conserved during the evolutionary transition from aquatic to terrestrial locomotion.

## CONFLICT OF INTEREST

The authors declare that they have no conflicts of interest to disclose.

## ROLE OF AUTHORS

All authors had full access to all the data in the study and take responsibility for the integrity of the data and the accuracy of the data analysis. Study concept and design: D.R., F.A., and R.D. Acquisition of data: D.R. and F.A. Analysis and interpretation of data: D.R., F.A., and R.D. Drafting of the article: D.R., F.A., and R.D. Critical revision of the article for important intellectual content: D.R., F.A., J.‐M.C., and R.D. Statistical analysis: D.R. Obtained funding: R.D. Administrative, technical, and material support: Danielle Veilleux. Study supervision: R.D.
